# Vegan versus meat-based cat food: Guardian-reported health outcomes in 1,369 cats, after controlling for feline demographic factors

**DOI:** 10.1371/journal.pone.0284132

**Published:** 2023-09-13

**Authors:** Andrew Knight, Alexander Bauer, Hazel Brown

**Affiliations:** 1 Centre for Animal Welfare, Faculty of Health and Wellbeing, University of Winchester, Winchester, United Kingdom; 2 School of Environment and Science, Nathan Campus, Griffith University, Nathan, QLD, Australia; 3 Statistical Consulting Unit StaBLab, Department of Statistics, LMU Munich, Munich, Germany; University of Life Sciences in Lublin, POLAND

## Abstract

Increasing concerns about environmental sustainability, farmed animal welfare and competition for traditional protein sources, are driving considerable development of alternative pet foods. These include raw meat diets, *in vitro* meat products, and diets based on novel protein sources including terrestrial plants, insects, yeast, fungi and potentially seaweed. To study health outcomes in cats fed vegan diets compared to those fed meat, we surveyed 1,418 cat guardians, asking about one cat living with them, for at least one year. Among 1,380 respondents involved in cat diet decision-making, health and nutrition was the factor considered most important. 1,369 respondents provided information relating to a single cat fed a meat-based (1,242–91%) or vegan (127–9%) diet for at least a year. We examined seven general indicators of illness. After controlling for age, sex, neutering status and primary location via regression models, the following risk reductions were associated with a vegan diet for average cats: increased veterinary visits– 7.3% reduction, medication use– 14.9% reduction, progression onto therapeutic diet– 54.7% reduction, reported veterinary assessment of being unwell– 3.6% reduction, reported veterinary assessment of more severe illness– 7.6% reduction, guardian opinion of more severe illness– 22.8% reduction. Additionally, the number of health disorders per unwell cat decreased by 15.5%. No reductions were statistically significant. We also examined the prevalence of 22 specific health disorders, using reported veterinary assessments. Forty two percent of cats fed meat, and 37% of those fed vegan diets suffered from at least one disorder. Of these 22 disorders, 15 were most common in cats fed meat, and seven in cats fed vegan diets. Only one difference was statistically significant. Considering these results overall, cats fed vegan diets tended to be healthier than cats fed meat-based diets. This trend was clear and consistent. These results largely concur with previous, similar studies.

## Introduction

Global pet food consumption is large and growing. By 2018, the pet population globally was estimated to include 471 million dogs, and 373 million cats [1, p. 4]. By 2018 the European pet cat population was estimated at 103.8 million, with 25% of households owning at least one cat. Additionally, many millions of additional dogs and cats who are not pets also consume some pet food, e.g., from cat colony feeders. By 2014, total pet food sales internationally were worth Euro 131.7 billion [2]. Driven primarily by pet food premiumisation and overall inflation, the UK pet food market was expected to reach GBP 2.9 billion by the end of 2020, having risen 16% since 2015 [3]. US pet food and treat sales were also rising, being worth USD 42.0 billion by 2020 [4].

A market of such size drives considerable research and product development, and between January 2013 and October 2014, over 6,000 new petfood products (3,000 dry and 3,200 wet pet foods), as well as 4,000 new pet snacks, were launched globally [5, 6]. Some of the new products being developed include raw meat diets, *in vitro* meat products, and diets based on novel protein sources, including terrestrial plants, insects, yeast, fungi and seaweed. Some of this development may be driven by significant recent concerns about the environmental sustainability of animal agriculture, and of traditional pet foods based on animal produce [7–11].

However, concerns exist that the imposition of human petfood preferences may be suboptimal for the welfare of cats. These concerns have been voiced by veterinary professional associations. As recently as 2020 the British Veterinary Association claimed that, “Cats are obligate carnivores and should not be fed a vegetarian or vegan diet. While on paper a diet may include supplements or alternatives to animal-based protein, there is no evidence these would be bioavailable to the cat or that they wouldn’t interfere with the action of other nutrients” [12]. Evidence concerning ingredient bioavailability and interactivity can indeed be lacking, but to our knowledge there is no published evidence that such concerns are any greater for non-animal-based ingredients, than for animal-based ingredients. Going even further, Loeb [13] claimed (albeit also without evidence) that “… an owner who feeds his or her cats a vegan diet … could be committing a crime under the Animal Welfare Act …”, and has repeated similar claims elsewhere [12].

How hazardous for cats are vegan diets? There are two obvious ways to assess the nutritional soundness of such diets for cats. The first involves examining steps taken by petfood manufacturers to ensure the quality and nutritional soundness of their products. These were recently examined in a survey of 29 companies producing meat-based (19) and plant-based (10) pet foods [14]. Although there were limited areas in which practices could be improved, most manufacturers had acceptable or superior standards at nearly all stages examined, throughout the design, manufacturing, transportation and storage phases, with plant-based diets slightly superior to meat-based diets overall.

However, the most important test is always the effects on the animals themselves. This is why feeding trials are considered the gold standard to ensure nutritional soundness of new formulations [15, 16]. The health status of cats maintained on different diets has been the subject of limited studies to date. In 2021 Dodd *et al*. [17] published a Canadian-based survey of 1,325 cat guardians, of whom 1,026 described their cat(s) diet. These included 187 (18%) vegan cats. More guardians of vegan cats reported their cat to be in very good health, and fewer were reported to have gastrointestinal and hepatic disorders. These cats were more often reported as having ideal body condition scores, than those fed a meat-based diet.

Several prior studies have been conducted, which we have reviewed elsewhere [18]. In 2014, Semp [19] reported the results of a study of vegan companion animals in Austria, Germany and Switzerland. A questionnaire completed by 59 cat (and 174 dog) guardians who were feeding a vegan diet revealed that participating cats had eaten vegan diets for six months to 6.5 years, with a mean of 3.9 years. Thirty-eight of these cat and dog guardians reported healthier and shinier coats after transitioning to vegan diets. Some showed resolution of dermatological problems. Sixteen guardians described improved odours of their pets. Some also noted increased stool volumes and improvement of stool consistency.

Semp’s questionnaire was followed by a clinical examination and blood tests on 15 cats (as well as 20 dogs), all randomly selected. Twelve (80%) had an ideal body weight, with three (20%) being overweight. Twelve (80%) had a normal, shiny coat. Three (20%) had signs of dandruff. Other than one cat with flea allergy dermatitis, none had pruritic (itchy) skin. Other clinical signs were virtually all normal. Haematological (complete blood count) and biochemical (liver, kidney, and pancreatic) parameters were assessed, as well as levels of magnesium, calcium, iron, total protein, folic acid, vitamin B12, and carnitine. During standardized clinical examinations, no abnormalities were detected that were associated with diet. When considering blood test results, serum total protein of all 15 cats and 20 dogs studied were within normal ranges. For the cats, the main abnormality observed was significantly lower folic acid (vitamin B9) values in vegan cats, compared to conventionally fed cats. Semp stated that, “The reason… is not known and may need further investigation”. In cats, folate deficiency is associated with hyperhomocysteinaemia (increased blood homocysteine levels) [20]. Homocysteine levels depend on the methionine metabolic cycle [21, 22]. Demethylation of methionine produces homocysteine. Hyperhomocysteinaemia may be associated with thromboembolic disease, although this is not described as an important risk factor [23]. Metabolic pathways that reduce homocysteine levels require adequate levels of vitamins B6, B9 and B12 [24]. No other significant deviations from normal values were observed. In particular, lower values of iron, protein or vitamin B12 in vegan cats were not observed. For the dogs, no significant differences were evident in any of the tested parameters, compared to the dogs fed a conventional diet.

In 2006, Wakefield and colleagues [25] published the first study of the health of a population of cats maintained on vegetarian diets (most, in fact, were vegan), long-term. Thirty-four cats were maintained on vegetarian diets and 52 on conventional diets, for at least one year. No significant differences existed between the two groups in age, sex, body condition, housing, or perceived health status. Most of the caregivers in both groups described their cats as healthy or generally healthy. Blood taurine and cobalamin (vitamin B12) levels were also assessed for 17 of these cats that had exclusively been fed either a commercial or a homemade vegetarian diet. Cobalamin levels were within the normal range in all cases, and taurine levels were similarly normal in 82% (14/17) of cases. The remaining three cases were cats who were partly maintained on dinner table scraps. Because such scraps are not nutritionally complete or balanced, these should always comprise a minority of diets.

Within a study of 86 vegetarian dogs and eight vegetarian cats in Germany, Switzerland, and Belgium, published in 2001, Kienzle and Engelhard [26] found numerous dietary deficiencies. Surprisingly perhaps, no clinical problems were found in the adult dogs. However, one cat showed symptoms of retinal atrophy, and two displayed reduced frequency of oestrus. In 1992, Leon and colleagues [27] confirmed that cats maintained on nutritionally deficient diets may experience health problems. In this case, the vegetarian diet studied was formulated to be deficient in potassium, and clinical signs involved neuromuscular function, which is known to be caused by potassium deficiency, among other possible causes [28, pp. 712–713].

However, these studies had various constraints which limit their predictive value for wider cat populations, and particularly for cats on vegan diets formulated to be nutritionally sound. In some cases, diets used in these studies were known to be nutritionally deficient. Blood tests were rarely comprehensive, and sample sizes were sometimes limited. By 2020, no large-scale study of cats had been published, describing how health indicators vary between cats maintained on vegan or meat-based diets. Accordingly, we designed a study to explore this. Our null hypothesis was that feline health indicators would not significantly vary with diet type. The success of new pet foods under development also depends on the views of consumers. We sought to determine the importance of pet health outcomes as a purchasing determinant, to a large group of cat guardians. Results of related survey parts were recently reported (palatability of different diets, finding no significant differences overall between vegan and meat-based cat food; [29]).

## Methodology

Details of our Methodology have been described elsewhere [30]. We designed a survey for cat or dog guardians using the ‘Online surveys’ platform (https://www.onlinesurveys.ac.uk). Guardians were asked to provide information about themselves and one cat or dog resident within their household for at least one year. Where animals were fed a prescription or therapeutic diet, guardians were asked to base answers on the diet in use prior to the commencement of the therapeutic diet. Guardians were asked about the main ingredients within their pet’s normal diet. They were asked to identify whether the diet was based on conventional, raw or *in vitro* meat, insects, fungi or algae, or whether it was a vegetarian, vegan or ‘other’ diet. Respondents could select only one option. Vegetarian diets were explained as including eggs or milk, but not meat, and vegan diets as eschewing any animal products. Guardians were also asked about any treats/snacks/scraps or supplements provided. We did not further inquire about details of diets, including nutritional soundness indicators, such as packaging claims of compliance with the nutritional guidelines of the European Pet Food Industry Federation (FEDIAF), or the Association of American Feed Control Officials (AAFCO).

Our survey also inquired about human demographics: continental region, urban or rural location, educational qualifications achieved, occupation, household income, age categories in 10 year age bands with the exception of bands for 18–19 and ‘> 70’, gender, and respondent diet. We also inquired about factors of importance to guardians when choosing pet food, and information sources guardians relied on. Information was also obtained about animal-related factors, including demographics. These included: role (companion or working animal), age (with any year entry up to ‘> 25’ possible), sex/neuter status, activity level, health status and reactions to meals.

Guardians provided information about seven general indicators of illness, and about the prevalence of specific health disorders, for the previous year, or the year prior to the commencement of a therapeutic diet, if one was currently used. Specifically, guardians were asked to report the frequency of veterinary visits, and of medication use (other than routine vaccinations and treatments for external or internal parasites, such as fleas, ticks, lice, heartworm and intestinal worms, or treatments associated with neutering operations or microchipping). Guardians were asked to report whether their cat had progressed onto a therapeutic diet, after initial maintenance on another diet type. They were asked to report their own opinion of their cat’s health status, and also to report what they believed their veterinarian’s health assessment to be. Guardians were asked to “Think about your veterinarian. Which of the following would most likely describe their opinions about your animal’s medical condition over the previous 12 months?” Possible answers ranged from no problems/routine preventative healthcare, to seriously ill. If veterinarians reportedly considered cats to be suffering from health disorder(s), guardians were asked which disorder(s) these were, from among 18 disorders indicated to be among the most common disorders experienced by companion cats [31–35]. Guardians were able to select multiple disorders, and to provide details of additional disorders by selecting ‘other’. Details for each ‘other’ entry were examined, with these entries then reclassified into 18 existing or four new disorder types, giving a total of 22 possible health disorders.

When analysing health disorders, cases were excluded, where veterinary visits had not occurred at least once in the previous year, or where guardians were unsure of the assessments of their veterinarians. The remaining subset comprised guardians who had recently seen their veterinarians, and were sure of their health assessments. This subset was used to calculate the proportion of unwell cats, and the average number of cases of health disorder, per unwell cat. It was also used to calculate the prevalence of the 22 specific health disorders.

### Potentially confounding factors

Health status may be affected by age, sex and desexing (neutering) status [34, 35]. Hence, we sought to ascertain differences between major dietary groups, in age, sex and neutering status. We decided not to attempt to account for the possible effects of certain additional factors, on health outcomes. Breed, for example, can affect also health status [36]. However, we were concerned that small numbers within breed groups would limit our ability to statistically analyse subsequent results, and so ultimately elected not to discriminate by breed within this study.

### Survey pilot and distribution

Our survey piloting and distribution were described in our related study on dietary palatability [29]. The ‘Online surveys’ platform we chose to use complies with the UK General Data Protection Regulation, following the UK Data Protection Act 2018, and was used by 88% of UK higher education institutions by 2019 [37], including our University of Winchester.

**Fig 1:**
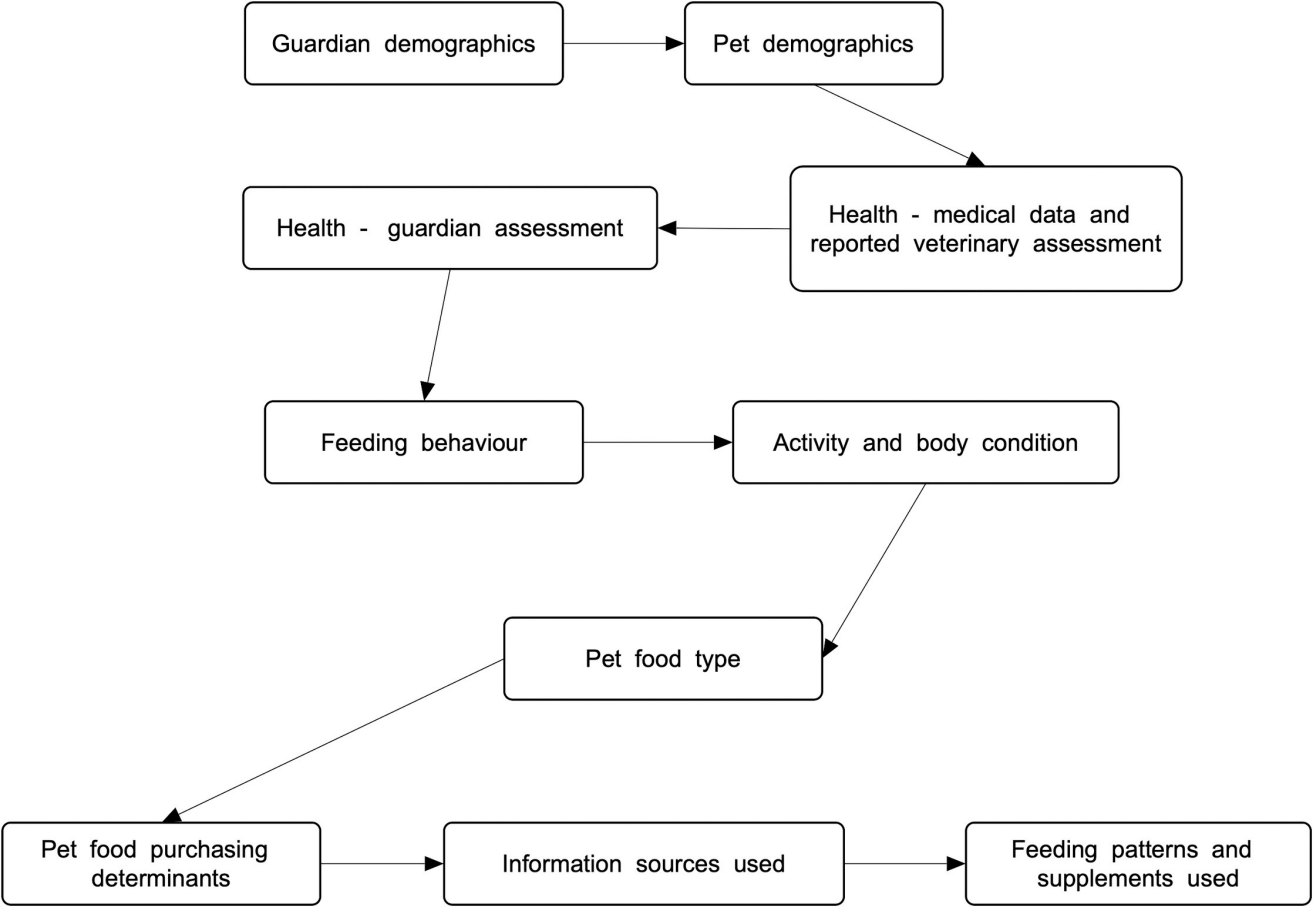
Survey parts. Source: [30].

We piloted our survey to 25 respondents in April 2020. Improvements were then made to both survey structure and questions. With respect to structure, changes were made to the ordering of survey parts, to minimise inadvertently biasing answers to questions about health. These survey sections were moved toward the beginning, to eliminate chances that answers might be affected by prior answers about pet diet choices, particularly where unconventional diets were used, e.g., if a guardian reporting use of an unconventional diet might subsequently be more likely to consciously or unconsciously downplay any health problems. Similarly, changes were made to the ordering of questions about veterinary opinions about animal health. In general, the variable most likely to be dependent, was positioned prior to any possibly corresponding independent variable. Various questions were also clarified and simplified. The final survey steps were those in Fig 1.

The final survey was made available from May–December 2020. It was widely advertised through social media to cat and dog interest groups. Paid Facebook advertising and several volunteers were utilised to increase survey exposure. Facebook advertising demographics were unlimited, other than to include terms relating to cats and dogs. In anticipation of lower levels of unconventional diets, and the need to achieve group numbers sufficient for statistical analysis, volunteers and the authors made some efforts to reach unconventional pet food interest groups, as well as conventional cat and dog interest groups. However, by careful wording choice, no bias for or against any particular diet was implied within advertising materials, nor within the survey questions or explanatory text.

### Statistical analysis

After initial examination of cat diets, further analysis was limited to cats maintained on two main diets: meat-based (conventional or raw), and vegan pet food. Initially, we examined the association of the two main diet types with feline demographic factors. Association with the categorical variables ‘primary location’, joint ‘sex and neuter status’, and each of ‘sex’ and ‘neuter status’ individually, were investigated using chi-square tests of independence [38]. For variables that showed significant differences, we provided effect size interpretations using the Cramer’s V statistic, with small, medium or large effects interpreted when V was close to 0.2, 0.5 and 0.8, respectively [39]. Differences between mean ages were explored using an independent samples t-test with unequal variances (“Welch test”, [38]). When significant differences in these mean ages were detected, effect size interpretations were provided using the Cohen’s d statistic, with small, medium and large effects interpreted when |d| was close to 0.2, 0.5 and 0.8, respectively [40].

Next, associations were investigated between the two main diet types and cat health. Guardians provided information about seven general indicators of illness, and about the prevalence of 22 specific health disorders. The seven general indicators of illness were: increased numbers of veterinary visits, medication use, progression onto a therapeutic diet after initial maintenance on a vegan or meat-based diet, reported veterinary assessment of being unwell, reported veterinary assessment and guardian opinion of more severe illness, and number of health disorders per unwell cat. The 22 specific disorders reflected veterinary assessments as reported by guardians.

Potential associations of the diet type with all seven indicators of illness were investigated using separate generalized additive regression models (GAMs; [41]). The estimation of additive regression has two main benefits compared to individual hypothesis tests. First, regression models allow adjustment for differences of the dietary groups in control variables, e.g., controlling for the fact that vegan diet cats in our study were on average 1.9 years younger than cats fed a meat-based diet. Second, additive regression allows for the estimation of nonlinear effects. In our models, the effect of age was estimated nonlinearly (based on a P-spline basis with four basis functions). This is necessary, since, e.g., the number of veterinary visits does not rise or decline by a constant factor every year, but follows a ‘U’ shape where very young and very old cats both have higher number of veterinary visits, independent of diet. All seven regression models included the same set of control variables: age, sex and neuter status, and the cat’s primary location (Table 1). For the categorical variables, we assigned the categories occurring most frequently, as the reference categories.

**Table 1:** Primary location of 1,368 cats fed meat-based or vegan diets. Note: Results are reported as ‘absolute frequency (relative frequency; standard error of the mean [in percentage points])’.

Primary location	Meat	Vegan	Total
Mostly indoor	705 (57%; SEM = 1.4PP)	85 (67%; SEM = 4.2PP)	790 (58%, SEM = 1.3PP)
Indoor and outdoor	491 (40%; SEM = 1.4PP)	37 (29%; SEM = 4.0PP)	528 (39%; SEM = 1.3PP)
Mostly outdoor	45 (4%; SEM = 0.5PP)	5 (4%; SEM = 1.7PP)	50 (4%; SEM = 0.5PP)
**Total**	**1,241 (100%)**	**127 (100%)**	**1,368 (100%)**

Depending on the variable type for each health indicator, different types of regression models were estimated (see [42] for all following types). Binary logistic regression was estimated for variables ‘increased numbers of veterinary visits (comparing ‘0–1 visits’ to ‘2 or more visits’), ‘medication use’, ‘progression onto a therapeutic diet’ and ‘reported veterinary assessment of being unwell’. Ordinal logistic regression was estimated for variables ‘reported veterinary assessment of more severe illness’ and ‘guardian opinion of more severe illness’ (comparing the two consecutive thresholds between the three categories ‘healthy’, ‘minor problems’ and ‘frequent problems/seriously ill’). Quasi-Poisson regression was estimated for variable ‘number of health disorders per unwell cat’, with an estimated dispersion parameter of 0.51.

Consistent with state-of-the-art statistical practice, our interpretations followed the American Statistical Association’s statement on the use of p-values [43, 44]. Accordingly, we used p-values and confidence intervals (CIs) with a significance level of 0.05 to evaluate the (un)certainty of the effects, but not as an evaluation of the “relevance” of effect sizes. The main focus should be on effect strength, measured based on the coefficient value, rather than on the p-value. Results were interpreted as “association” (when a significant effect ~10% or stronger), “strong tendency” (when a non-significant effect ~25% or stronger), “tendency” (when a non-significant effect ~10%—~25%) or “marginal” (when a non-significant effect <10%). In light of this, and given the number of comparisons associated with comparing the dietary groups, p-values were not corrected for multiple testing.

As well as exploring associations between dietary differences and the seven general health indicators, associations with the 22 specific disorders were explored. For each disorder, a separate additive logistic regression model was estimated, including age (nonlinear), sex and neuter status, and primary location, as linear control variables.

Goodness of fit was evaluated using area under the curve (AUC) values for (ordinal) logistic regression, and the explained share of deviance and the mean absolute error (MAE) for the Quasi-Poisson regression [42]. AUC and MAE values were calculated on a randomly drawn 20% test set, based on a re-estimation of the regression models on the respective 80% training set. AUC values were 0.58 (variable ‘increased numbers of veterinary visits’), 0.58 (‘medication use’), 0.59 (‘progression onto a therapeutic diet’), 0.66 (‘reported veterinary assessment of being unwell’), 0.64 and 0.64 (for the two thresholds for variable ‘reported veterinary assessment of more severe illness’), and 0.62 and 0.77 (for the two thresholds for variable ‘guardian opinion of more severe illness’). Based on AUC values for the first three models indicating a non-optimal fit, the respective results should be interpreted with some care. The same holds true for the Quasi-Poisson model for variable ‘number of health disorders per unwell cat’, with a 12% explained share of deviance and a MAE of 1.13.

All interpretations were based on a significance level of 0.05. All analyses were conducted with the open-source software R [45]. Regression model estimation was performed using function *gam* from R package *mgcv* [41].

### Ethical approval and data availability

Our research complied with the University of Winchester Ethics Policy [46] (approval reference RKEEC200304_Knight). Survey participants were initially informed about the survey purpose and questions. Each was then required to confirm in writing that they were “aged 18 or older and consent to participating”, and that their answers related to one dog or cat that had been within their household for at least one year, before gaining access to the survey questions. Our data analysed, along with the R code used for its statistical analysis, are accessible at https://osf.io/nbepu.

## Results

Of 4,060 respondents to our combined cat and dog survey, 4,057 confirmed they met the survey conditions (18 years or older, with answers relating to one dog or cat resident within their household, for at least one year). The following results are limited to the 1,418 cats and their guardians who responded. Results concerning 2,639 dogs and their guardians were reported in a related study [30].

### Cat guardians

Of the 1,399 human respondents who provided their sex, 91% (1,269) identified as females, 9% (124) as males, and 0% (6) as other. Most age brackets from 18 to 70+ were well represented, other than the extreme ends where numbers were low. The overwhelming majority of the 1,418 total respondents identified their geographical region as the UK (70%, 998) or Europe (20%, 279), with North America (5%, 68) and Australia/New Zealand/Oceania (3%, 43) being the next most prevalent continental regions. A minority (10%, 141) of the 1,418 total respondents worked in the pet or veterinary industries. These 1,418 respondents reportedly followed a variety of diets themselves, with the most common being omnivorous (35%, 500), vegan (26%, 372), reducetarian (omnivore reducing animal product consumption) (22%, 318), vegetarian (10%, 146) and pescatarian (consuming fish but no other meats) (5%, 72).

#### Importance of health to guardians

Of the 1,397 respondents who indicated their involvement in pet diet decision-making, 94% (1,318) were primary decision-makers, 4% (62) played some lesser role, and 1% (17) played no role. Those 99% (1,380) playing at least some role were asked which factors were important when choosing pet diets. Among 13 options including ‘other’, health and nutrition was considered the most important factor, being of importance to 85% (1,171) of 1,380 respondents to this question. These 1,171 individuals were asked which health and nutrition factors were important to them. Maintenance of pet health was considered the most important factor among five health and nutrition options including ‘other’. It was cited as important by 88% (1,028) of 1,170 respondents to this question.

The importance of health was similarly highlighted by the 1,178 respondents who used a conventional meat formulation as their cat’s normal diet, and the 64 who used a raw meat formulation. These combined 1,242 respondents were asked whether they would realistically choose alternative diets, if these offered their desired attributes and standards. The alternatives offered for consideration were vegetarian and vegan diets, as well as those based on laboratory grown meat, insects, fungi and algae. Of 1,227 who answered this question, 51% (630) confirmed they would realistically choose such alternative diets. ‘Confidence about pet health’ was the most important among 14 desired attributes (including ‘other’) that any alternative diet would need to provide. It was cited as essential by 83% (520) of these 630 respondents.

### Cats

#### Diets

**Fig 2:**
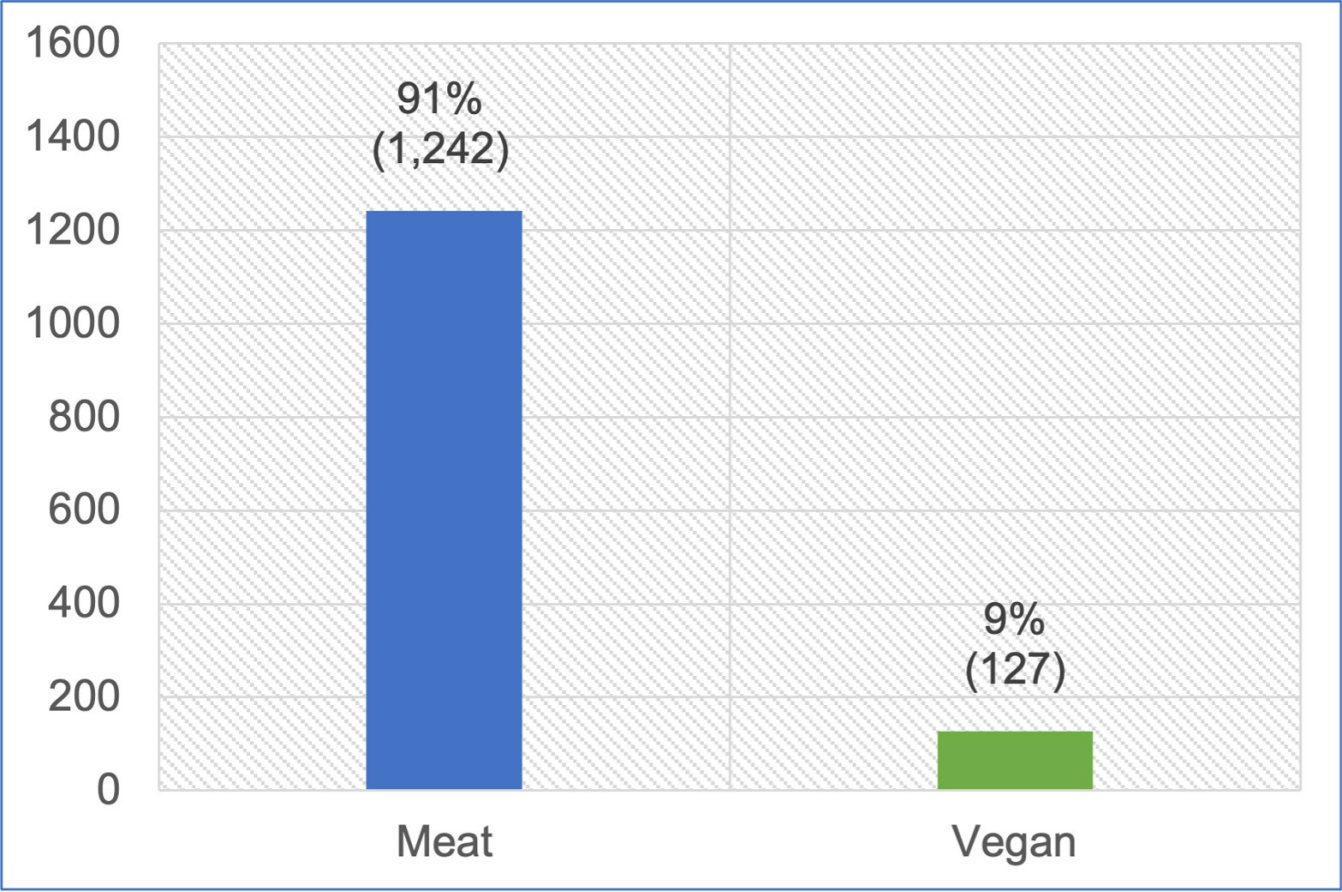
Meat-based or vegan diets fed to 1,369 cats.

In total 1,418 cat guardians responded, each describing a single cat. Of these, 1,397 indicated the main diet their cat was maintained on. Within this set, 1,369 cats were jointly maintained on the two main diets: 1,242 (91%) meat-based (conventional meat: 1,178 and raw meat: 64), and 127 (9%) on vegan diets (Fig 2). Thirty ’other’ diets were examined and reclassified into conventional meat, mixture or unsure, depending on further details provided in textual answers. Since the focus of our study was on conventional or raw meat-based and vegan diets used for bodily maintenance or growth, animals in some small, very specific dietary groups were excluded from further analyses. We excluded 28 animals reportedly maintained on an insect-based diet (1), vegetarian (3), laboratory-grown meat (9), mixtures of other dietary types (7) and diets listed as ‘unsure’ (8), due to low numbers, lack of clarity concerning main ingredient type, or current unavailability of these sources as pet maintenance diets (as distinct from treats, snacks or supplements).

A minority (41% - 558/1,369) of these respondents stated they provided treats/snacks/scraps at least once daily. Treats provided to these 1,369 cats were most commonly commercial treats (705), human food prepared at home (312), dental/oral bars or chewable sticks (311), and human food from other sources (108). Less common treats included raw meat or bones (77), and vegetables or fruit (72).

Thirteen percent (184) of these 1,369 cats were also regularly offered dietary supplements other than treats/snacks/scraps. These included vitamins (56), products for joint health (48), fatty acids (e.g., omega-3 fatty acids) (42), probiotics or prebiotics (41), amino acids (39) such as taurine, minerals (35), digestive enzymes (22), and other products.

#### Primary location

**Fig 3:**
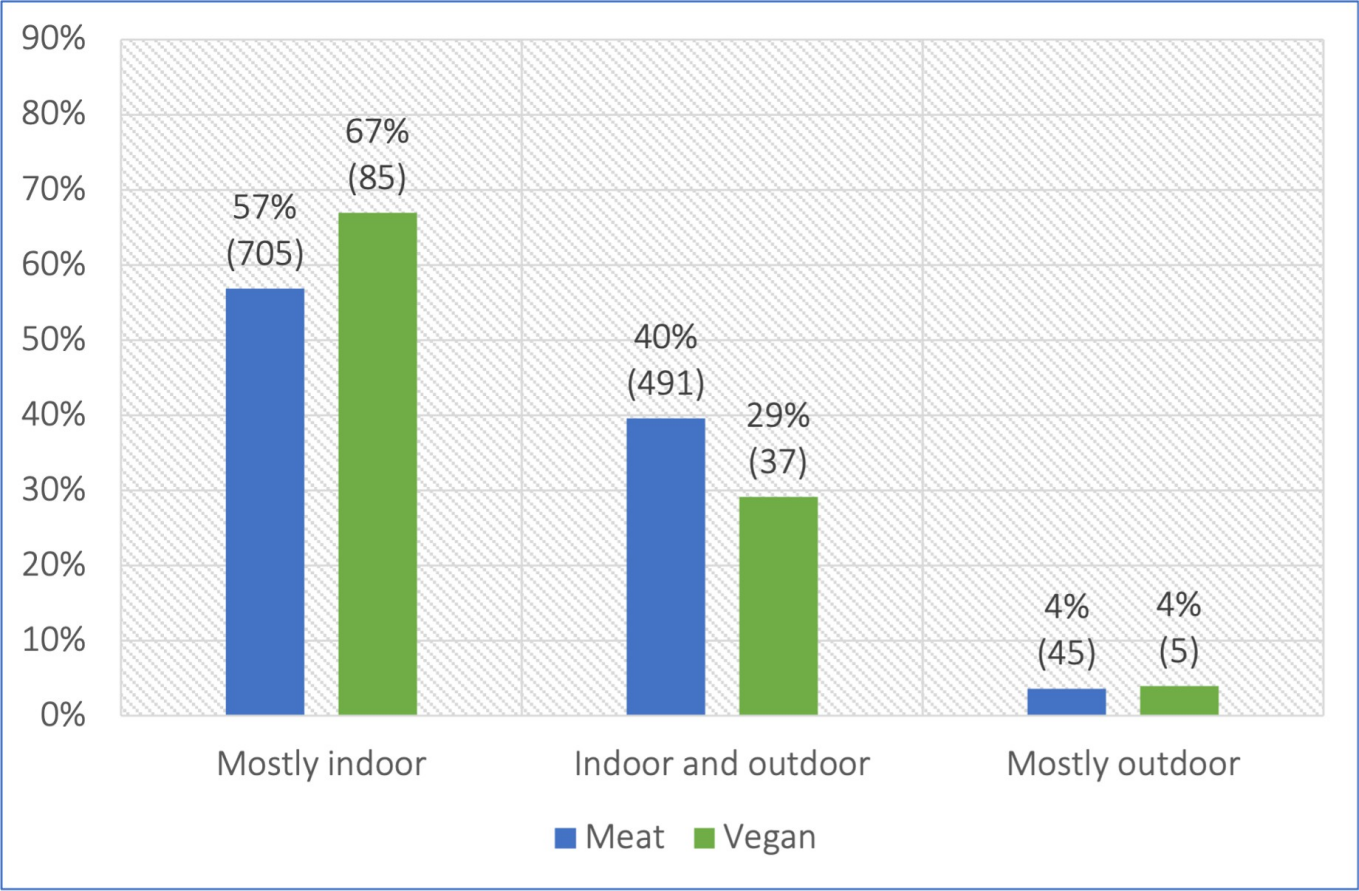
Primary location of 1,368 cats fed meat-based or vegan diets.

One guardian was unsure of the primary location of their cat. Fifty-eight percent of the other 1,368 cats mostly stayed indoors. This was 57% and 67% for cats fed meat-based and vegan diets respectively. The remaining cats were reported to stay mostly outdoors, or to spend significant time in both locations (Fig 3, Table 1). A chi-square test of independence indicated no significant association between diet and primary location (p = 0.0699).

#### Ages

**Fig 4:**
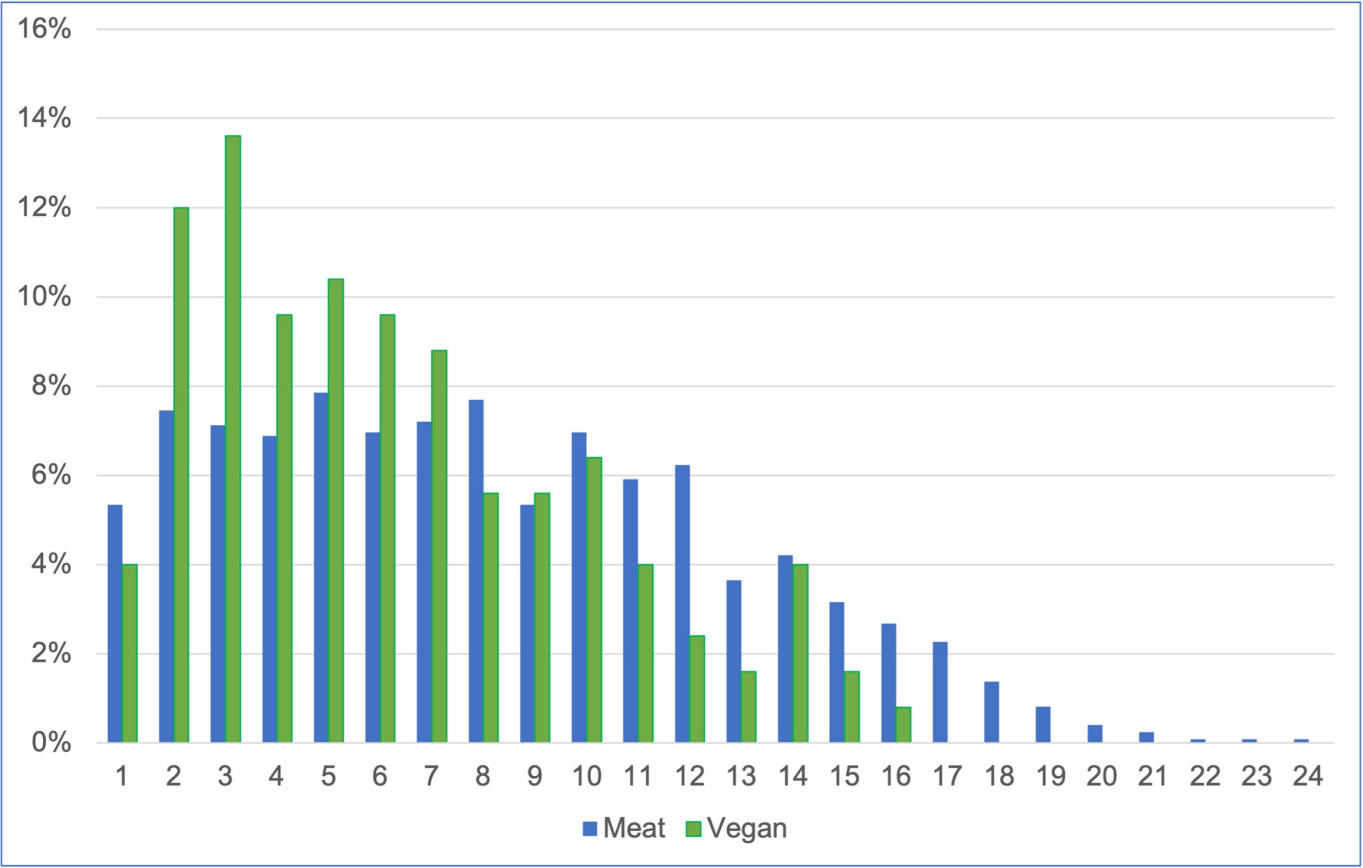
Ages of 1,360 cats fed meat-based or vegan diets.

Considering the 1,369 cats fed the two main diets, guardians were unsure of age in seven cases, and in two cases reported ages > 25 (31 and 49). Ages of the remaining 1,360 cats are provided in Fig 4.

The mean age of these 1,360 cats was 8.0 years overall. An independent samples Welch test was conducted to compare the ages of cats fed meat-based and vegan diets. There was a significant difference in mean ages, with vegan cats being, on average, 1.90 years younger (p < 0.001). Mean ages were: meat-based (M = 8.14, SD = 4.75, SEM = 0.14), and vegan diet (M = 6.24, SD = 3.72, SEM = 0.33). Cohen’s d = 0.44, indicating a medium effect size.

#### Sex/Neuter status

**Fig 5:**
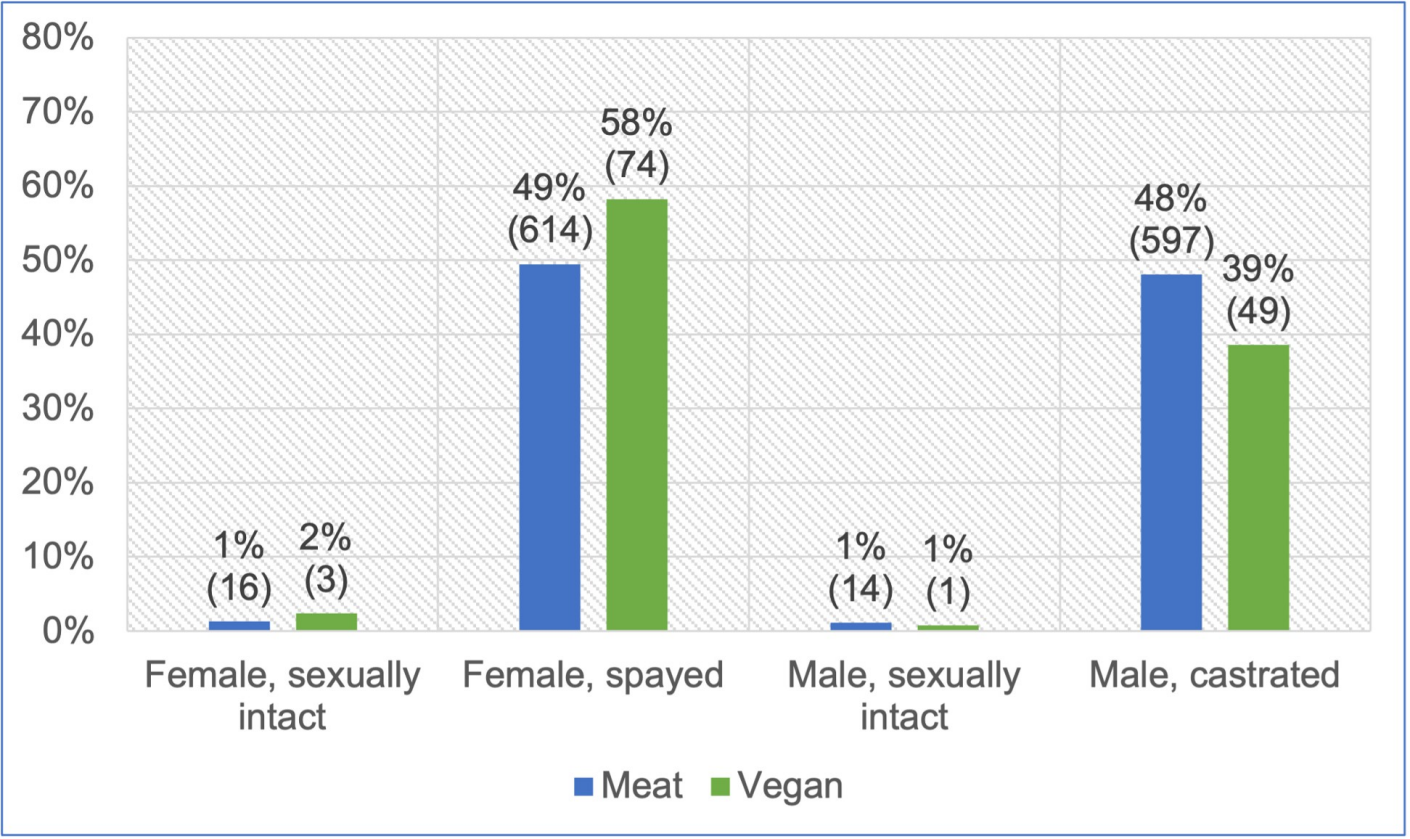
Sex/neuter status of 1,368 cats fed meat-based or vegan diets.

Considering the 1,369 cats fed the two main diets, guardians were unsure of sex/neuter status in one case. The sex/neuter status of the remaining 1,368 cats is provided in Fig 5 and Table 2. Females comprised 52% and males 48% of this sample. A chi-square test of independence showed no association between diet type and joint sex/neuter status (p = 0.1674).

**Table 2:** Sex/neuter status of 1,368 cats fed meat-based or vegan diets. Note: Results are reported as ‘absolute frequency (relative frequency; standard error of the mean [in percentage points])’.

Sex/neuter status	Meat	Vegan	Total
Female, sexually intact	16 (1%; SEM = 0.3PP)	3 (2%; SEM = 1.3PP)	19 (1%; SEM = 0.3PP)
Female, spayed	614 (49%; SEM = 1.4PP)	74 (58%; SEM = 4.4PP)	688 (50%; SEM = 1.4PP)
Male, sexually intact	14 (1%; SEM = 0.3PP)	1 (1%; SEM = 0.8PP)	15 (1%; SEM = 0.3PP)
Male, castrated	597 (48%; SEM = 1.4PP)	49 (39%; SEM = 4.3PP)	646 (47%; SEM = 1.3PP)
**Total**	**1,241 (100%)**	**127 (100%)**	**1,368 (100%)**

Considering sex individually, cats fed vegan diets were 60%:40% female:male respectively, whilst those fed meat-based diets were almost equally split. Sex and diet type were statistically significantly associated (p = 0.0428). The effect size was small (Cramer’s V = 0.057). Considering neutering status individually, almost all cats were neutered, and neutering status did not vary significantly with diet group (p = 0.8371).

### General indicators of illness (7): Effects of all control variables

The following results consider the 1,369 cats fed meat-based or vegan diets. The results of the regression models were interpreted based on the estimated exponentiated effects, as listed in Table 3. For the applicable variables, the average or most common observations respectively were: ‘meat-based diet’, ‘average age’ (~ 8 years; for models 3 and 4 (only) with linear age effects), ‘female, spayed’, and ‘mostly indoor habitat’. These were assigned as the reference categories for these variables (as indicated in Table 3). The exponentiated intercept parameters in Table 3 encode the odds (models 1–6) of the respective ‘unhealthy’ outcomes occurring, or the expected number of disorders (model 7), for cats with these reference characteristics. Models 3–4 had linear age effects, but age effects in the remaining models 1–2 and 5–7 were nonlinear, as visualised in S2–S6 Figs. The reference ages for the intercepts in these models was not ~8 years, but can be determined by reading the age values where the nonlinear effect curves in S2–S6 Figs cross the value of 1.0 (the dashed horizontal line, indicating no effect). For example, the intercept for model 1 (S2 Fig) refers to cats aged ~3 or ~12 years. In this case model 1 has two reference ages. However, the other models visualised each have a single reference age.

**Table 3:** Regression model results for seven general indicators of illness among 1,369 cats fed meat-based or vegan diets. Note: Cat numbers in some groups were lower than 1,369, as described under Results. The effect of each variable is reported as ‘exponentiated effect estimate [exponentiated 95% confidence interval], p-value’. Boldface indicates p < 0.05. The reference categories for variables ‘sex and neuter’ and ‘habitat’ are ‘female, spayed’ and ‘mostly indoor habitat’, respectively. The exponentiated intercept parameters encode the odds (models 1–6) of ‘unhealthy’ outcomes occurring, or the expected number of disorders (model 7), for cats with these reference characteristics. The effects of other categories state average differences compared to these reference categories. For the logistic regression models (models 1–6) these exponentiated effect estimates are odds ratios. For Quasi-Poisson model 7 the effects correspond to multiplicative changes in the expected number of health disorders per unwell cat. Some age effects were estimated nonlinearly. For these effects, the estimated degrees of freedom (edf) values are given (as a measure of how much each effect deviates from linearity), instead of a linear effect estimate. Visualizations of all control variable effect estimates and the nonlinear age effects can be found in S1–S6 Figs.

Parameter	Model 1 –increased vet visits	Model 2 –medication use	Model 3 –therapeutic diet	Model 4 –unwell	Model 5 –greater illness (vet assessment)	Model 6 –greater illness (guardian opinion)	Model 7 –no. of disorders per unwell cat
**Intercept**	0.448 [0.369–0.544]	0.392 [0.322–0.477]	0.077 [0.054–0.109]	0.856 [0.689–1.063]	0.311 [0.253–0.382]	0.248 [0.207–0.298]	1.650 [1.514–1.797]
**Vegan diet**	0.897 [0.588–1.368], p = 0.6133	0.804 [0.520–1.242], p = 0.3248	0.435 [0.156–1.216], p = 0.1126	0.936 [0.583–1.503], p = 0.7836	0.903 [0.570–1.430], p = 0.6621	0.731 [0.480–1.114], p = 0.1446	0.845 [0.672–1.064], p = 0.1525
**Age**	edf = 2.9, **p<0.0001**	edf = 2.5, **p = 0.0001**	1.088 [1.044–1.134], **p = 0.0001**	1.121 [1.089–1.154], **p<0.0001**	edf = 1.5, **p<0.0001**	edf = 1.9, **p<0.0001**	edf = 1.4, **p<0.0001**
**‍Female, spayed**	Reference category						
**Female, sexually intact**	0.431 [0.122–1.525], p = 0.1918	0.977 [0.339–2.815], p = 0.9661	0.960 [0.120–7.671], p = 0.9691	1.474 [0.392–5.538], p = 0.5656	1.202 [0.349–4.134], p = 0.7706	0.724 [0.241–2.175], p = 0.5645	1.067 [0.622–1.830], p = 0.8148
**Male, castrated**	1.172 [0.923–1.490], p = 0.1929	1.306 [1.028–1.659], **p = 0.0288**	1.669 [1.103–2.526], **p = 0.0154**	0.927 [0.707–1.216], p = 0.5853	1.009 [0.778–1.309], p = 0.9463	1.196 [0.949–1.506], p = 0.1291	1.042 [0.931–1.167], p = 0.4708
**Male, sexually intact**	0.406 [0.088–1.863], p = 0.2460	0.225 [0.029–1.738], p = 0.1527	1.547 [0.194–12.329], p = 0.6805	0.528 [0.107–2.613], p = 0.4336	0.522 [0.107–2.545], p = 0.4213	0.646 [0.177–2.354], p = 0.5073	0.740 [0.273–2.007], p = 0.5546
**‍Mostly indoor habitat**	Reference category						
**Indoor and outdoor habitat**	0.804 [0.625–1.034], p = 0.0894	0.959 [0.747–1.232], p = 0.7452	0.590 [0.376–0.927], **p = 0.0221**	0.648 [0.489–0.860], **p = 0.0026**	0.622 [0.473–0.818], **p = 0.0007**	0.592 [0.464–0.755], **p<0.0001**	0.868 [0.766–0.984], **p = 0.0280**
**Mostly outdoor habitat**	0.940 [0.497–1.778], p = 0.8486	1.134 [0.607–2.117], p = 0.6939	0.658 [0.198–2.191], p = 0.4954	1.327 [0.628–2.803], p = 0.4590	1.320 [0.648–2.686], p = 0.4446	0.575 [0.300–1.104], p = 0.0963	0.671 [0.469–0.962], **p = 0.0303**

For all regression models, results can be interpreted as the multiplicative change of the outcome, independent of the exact values of all control variables (which were diet, age, sex, neuter status and primary location). For the binary logistic models, the exponentiated effects are odds ratios (ORs), referring to a multiplicative change in the odds of the ‘unhealthy’ outcome occurring. For example, the estimated odds ratio of 0.897 for a vegan diet in the increased veterinary visits model indicates that cats fed a vegan diet had 100% x (1–0.897) = 10.3% lower odds of having two or more veterinary visits in the last year (an indicator of potential illness), compared to meat-fed cats, independent of any differences in the remaining control variables between the two dietary groups. For the ordinal logistic models, this multiplicative change refers to a change in the odds of being in a higher category (indicating more severe illness), compared to lower categories. For the Quasi-Poisson model, the exponentiated effects refer to a multiplicative change in the expected number of health disorders per unwell cat.

Exponentiated 95% confidence intervals are provided as measures for uncertainty. These confidence intervals are–by construction–not centred. While the confidence intervals for the raw linear effect estimates are centred, this centring is lost through the exponentiation step which enables the interpretation of the effects as odds ratios. For example, consider the effect of the vegan diet in model 5 for the ’reported veterinary assessment of more severe illness’. The raw effect estimate is -0.103 [CI: -0.563 to 0.358] which is centred. Only when exponentiating does the confidence interval become non-centred. When reporting as a multiplicative change in the outcome this becomes 0.903 [CI: 0.570 to 1.430] (Table 3). When reporting as an odds reduction with the vegan diet, this becomes -9.7% [CI: -43.0% to 43.0%] (Table 4).

**Table 4:** Effects on seven general indicators of illness of a vegan diet, in comparison to a meat-based diet, among 1,369 cats, after controlling for feline demographic factors. Note: Cat numbers in some groups were lower than 1,369, as described under Results. Results are reported as ‘exponentiated effect estimate [exponentiated 95% confidence interval]’, where negative percentages in a confidence interval refer to increases rather than reductions with a vegan diet. These exponentiated effects refer to reductions in the odds of six illness indicators occurring, or to reductions in the average number of health disorders per unwell cat. Results are interpreted as “association” (a significant effect ~10% or stronger), “strong tendency” (a non-significant effect ~25% or stronger), “tendency” (a non-significant effect ~10%—~25%) or “marginal” (a non-significant effect <10%) [43, 44].

Illness indicators (7)	Reduction with vegan diet	Interpretation			
		Marginal	Tendency	Strong tendency	Association
Increased veterinary visits	10.3% [-36.8% to 41.2%]		X		
Medication use	19.6% [-24.2% to 48.0%]		X		
Progression onto therapeutic diet	56.5% [-21.6% to 84.4%]			X	
Reported veterinary assessment of being unwell	6.4% [-50.3% to 41.7%]	X			
Reported veterinary assessment of more severe illness	9.7% [-43.0% to 43.0%]		X		
Guardian opinion of more severe illness	26.9% [-11.4% to 52.0%]			X	
No. of health disorders per unwell cat	15.5% [-6.4% to 32.8%]		X		
**Totals**		**1**	**4**	**2**	**0**

### General indicators of illness (7): Effects of diet

**Fig 6:**
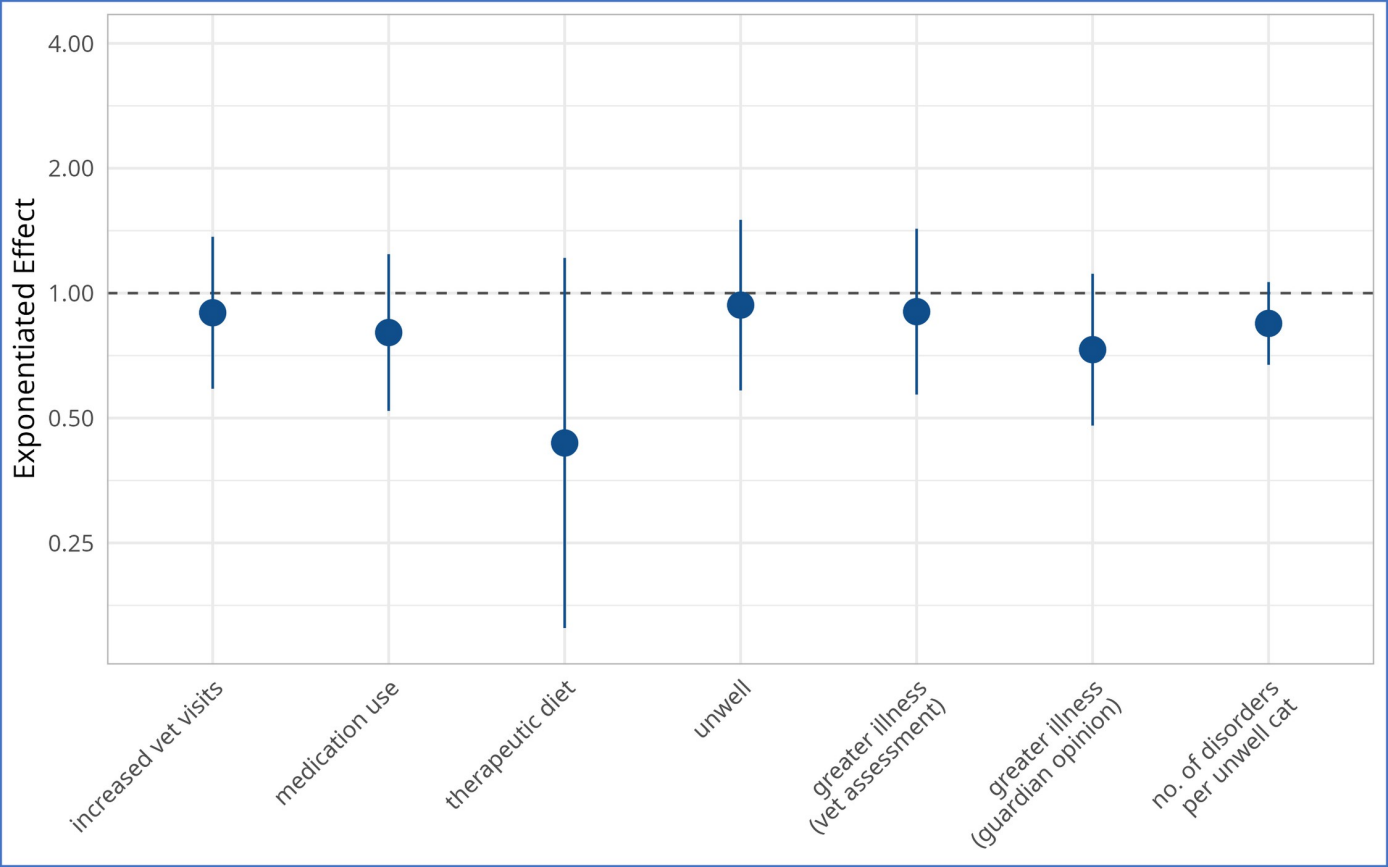
Exponentiated effect estimates for the ‘vegan diet’ coefficient, for all seven regression models, on a logarithmic y-axis, among 1,369 cats fed meat-based or vegan diets. Note: Cat numbers in some groups were lower than 1,369, as described under Results. Blue dots mark the exponentiated effect estimates. Blue bars mark the corresponding exponentiated 95% confidence intervals. All estimates are listed in Table 3.

The effects of the vegan diet on all seven general indicators of illness are visualized in Fig 6 and summarised in Table 4.

These results were then applied to an average cat with the reference characteristics described previously. For such a cat, relative reductions of occurrences of the seven general indicators of illness following a vegan diet are given in Table 5. For example, given the estimated results for model 3, an average cat with the reference characteristics had odds of 0.077 of having progressed onto a therapeutic diet in the last year (Table 3). For an average cat fed a vegan diet, the odds of having progressed onto a therapeutic diet reduced by 56.5% (Table 4), or by a factor of 0.435 (Table 3), to 0.033. Because these odds are defined as ‘Odds(‘therapeutic diet’) = P(‘therapeutic diet’) / (1 –P(‘therapeutic diet’))’, with P(‘therapeutic diet’) being the probability of having progressed onto a therapeutic diet, the corresponding probability can be calculated as ‘P(‘therapeutic diet’) = 100% x Odds(‘therapeutic diet’) / (1 + Odds(‘therapeutic diet’))’. Accordingly, for an average cat with the reference characteristics (including a meat-based diet), P(‘therapeutic diet’) = 7.1%. For a similar cat following a vegan diet, this fell to 3.2%–a relative reduction of 54.7% when exact (rather than rounded) figures were used.

**Table 5:** Prevalence of seven general indicators of illness among 1,369 cats fed vegan or meat-based diets, after controlling for feline demographic factors. Note: Cat numbers in some groups were lower than 1,369, as described under Results. Average cats were those with the reference categories defined previously.

Illness indicators (7)	Occurrence in average meat-based cat	Occurrence in average vegan cat	Relative reduction with vegan diet
Increased veterinary visits	30.9%	28.7%	7.3%
Medication use	28.2%	24.0%	14.9%
Progression onto therapeutic diet	7.1%	3.2%	54.7%
Reported veterinary assessment of being unwell	46.1%	44.5%	3.6%
Reported veterinary assessment of more severe illness	23.7%	21.9%	7.6%
Guardian opinion of more severe illness	19.9%	15.3%	22.8%
No. of health disorders per unwell cat	1.650	1.394	15.5%

#### Increased veterinary visits

**Fig 7:**
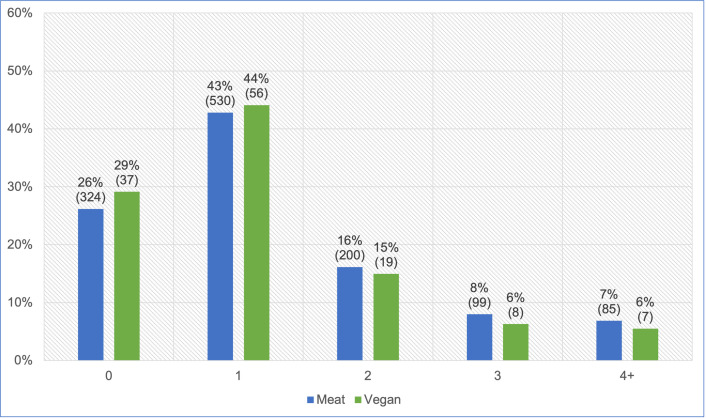
Veterinary visits of 1,365 cats fed meat-based or vegan diets, in the last year.

After excluding four ‘unsure’ responses, 1,365 guardians reported the frequency of veterinary visits within the last year (Fig 7). Routine health checks are normally conducted annually, whereas multiple veterinary visits may indicate a health problem. We were interested in those cats who saw veterinarians more than once in the previous year. When comparing two groups of 0–1 visits and 2 or more visits and controlling for cat demographic factors in the regression model, there was no significant association between veterinary visits and diet type (p = 0.6133). However, cats fed a vegan diet had, on average, 10.3% lower odds of having two or more veterinary visits (potentially indicating illness), compared to cats fed meat. Because the effect was stronger than 10%, but not statistically significant, it can be considered a *tendency*. For an average cat, this represented a 7.3% reduction in the risk of having two or more veterinary visits.

#### Medication use

**Fig 8:**
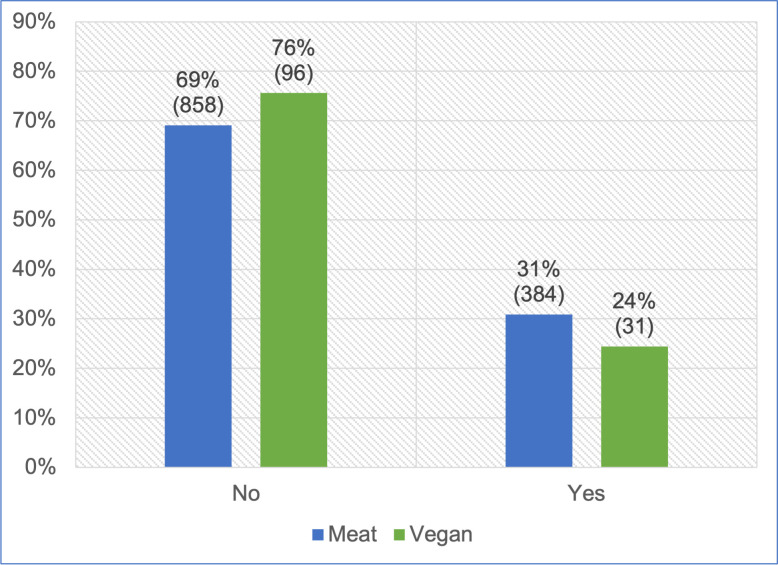
Medication use in 1,369 cats fed meat-based or vegan diets.

All 1,369 guardians provided information about medication use during the previous year (Fig 8). After controlling for cat demographic factors in the regression model, there were no significant differences in medication use between cats fed meat-based and vegan diets (p = 0.3248). However, cats fed a vegan diet had, on average, 19.6% lower odds of receiving medication (potentially indicating illness), compared to cats fed meat. Because the effect was stronger than 10%, but not statistically significant, it can be considered a *tendency*. For an average cat, this represented a 14.9% reduction in the risk of receiving medication.

#### Progression onto a therapeutic diet

**Fig 9:**
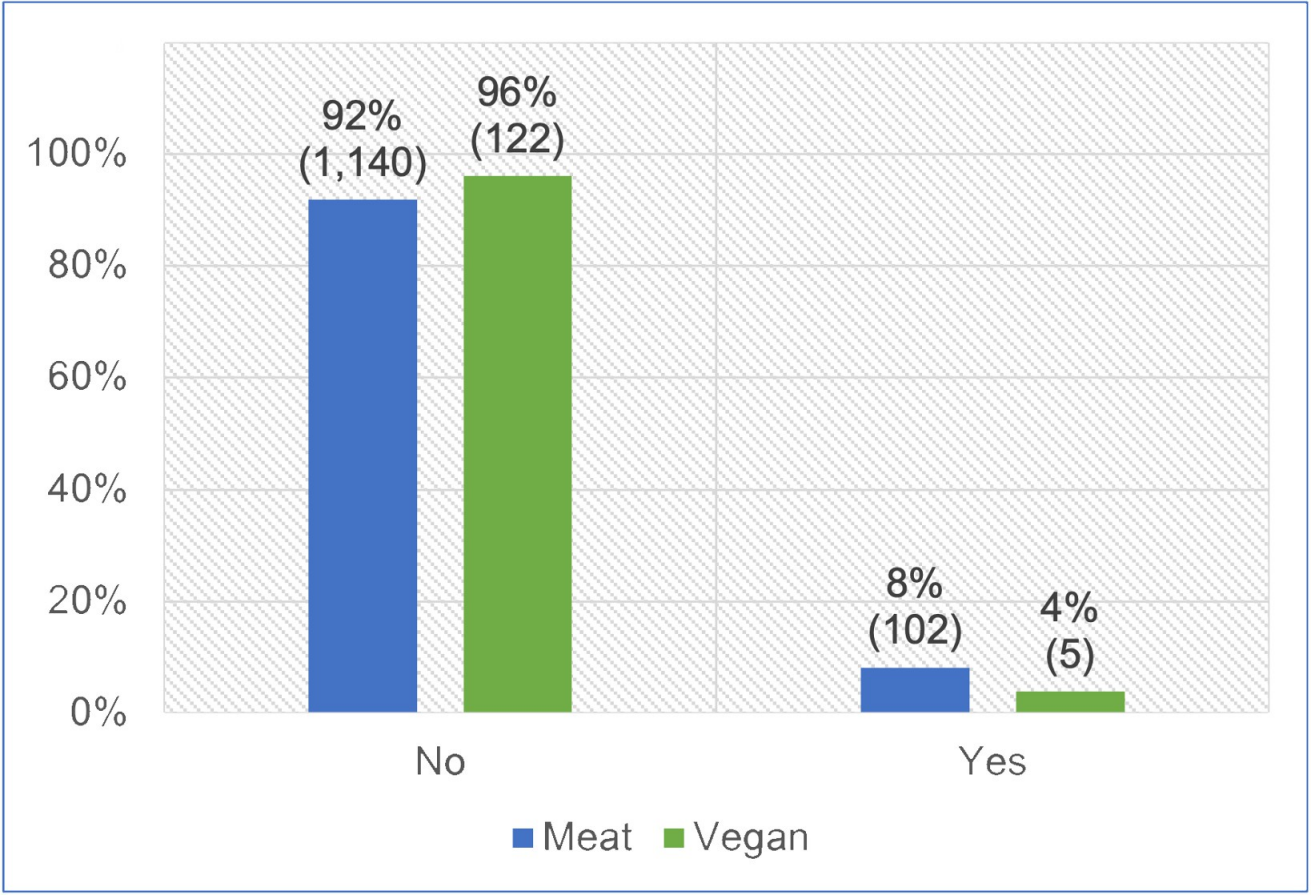
Subsequent progression onto a therapeutic diet in 1,369 cats maintained on an initial diet as specified.

All 1,369 guardians provided information about whether or not their cat progressed onto a therapeutic diet, after using one of the two main diets (Fig 9). After controlling for cat demographic factors in the regression model, there was no significant association in progression to a therapeutic diet between cats fed meat-based and vegan diets (p = 0.1126). However, cats initially fed a vegan diet had, on average, 56.5% lower odds of progressing onto a therapeutic diet (potentially indicating illness), compared to cats fed meat. Because the effect was stronger than 25%, but not statistically significant, it can be considered a *strong tendency*. For an average cat, this represented a 54.7% reduction in the risk of progressing onto a therapeutic diet.

#### Reported veterinary assessment of more severe illness

**Fig 10:**
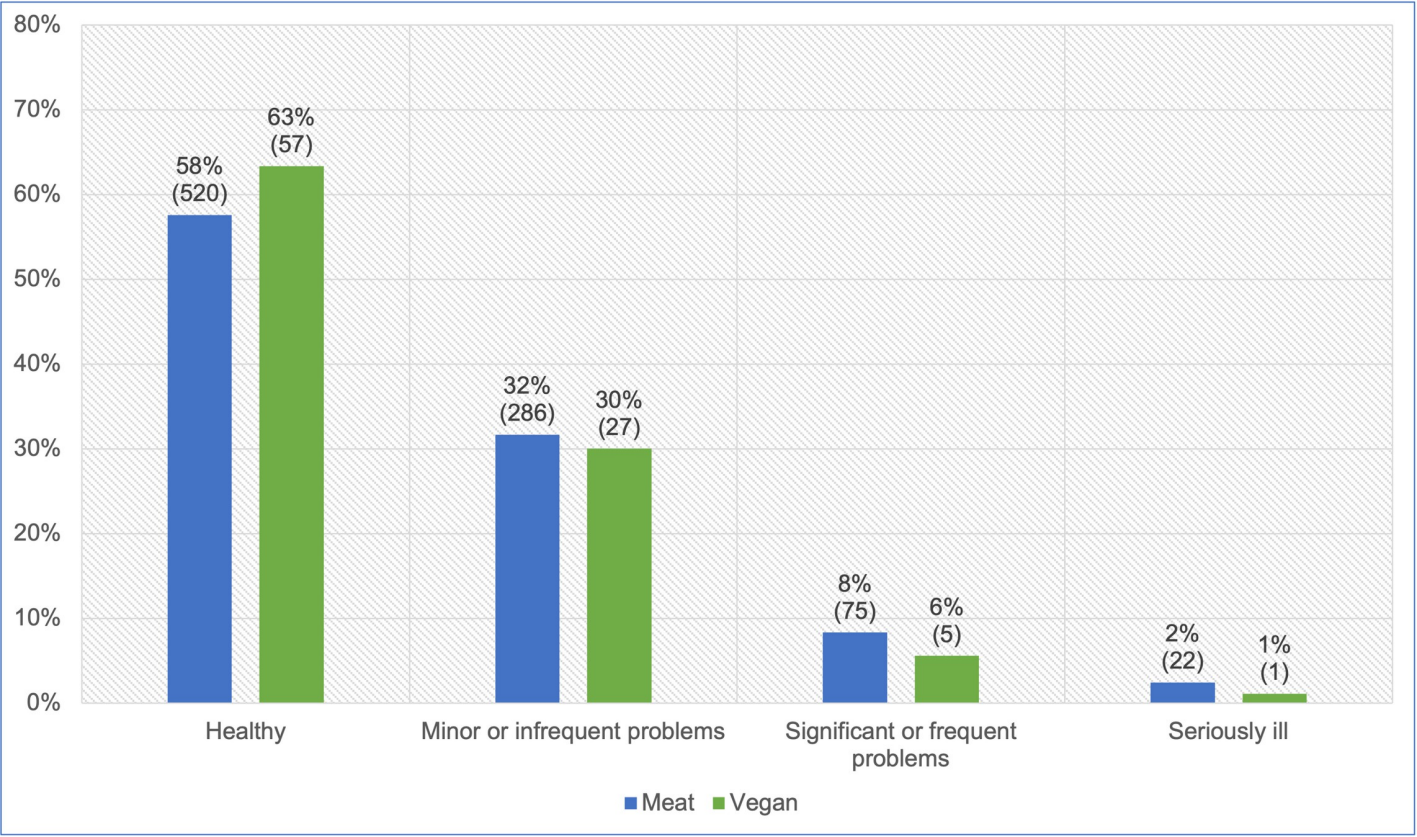
Reported veterinary assessments of illness severity of 993 cats fed meat-based or vegan diets.

A total of 1,004 cats saw a veterinarian at least once in the previous year. After excluding 11 ‘unsure’ cases, 993 of these guardians were reportedly sure of their veterinarians’ assessments regarding the illness severity of their cats (Fig 10). After controlling for cat demographic factors in the regression model, there were no significant differences in the reported veterinary assessments of illness severity between cats fed meat-based and vegan diets (p = 0.6621). However, cats fed a vegan diet had, on average, 9.7% lower odds of reportedly being considered by veterinarians to have more severe illness, compared to cats fed meat. Because the effect was very close to 10%, but not statistically significant, it can be considered a *tendency*. For an average cat, this represented a 7.6% reduction in the risk of reportedly being considered by veterinarians to have more severe illness.

#### Guardian opinion of more severe illness

**Fig 11:**
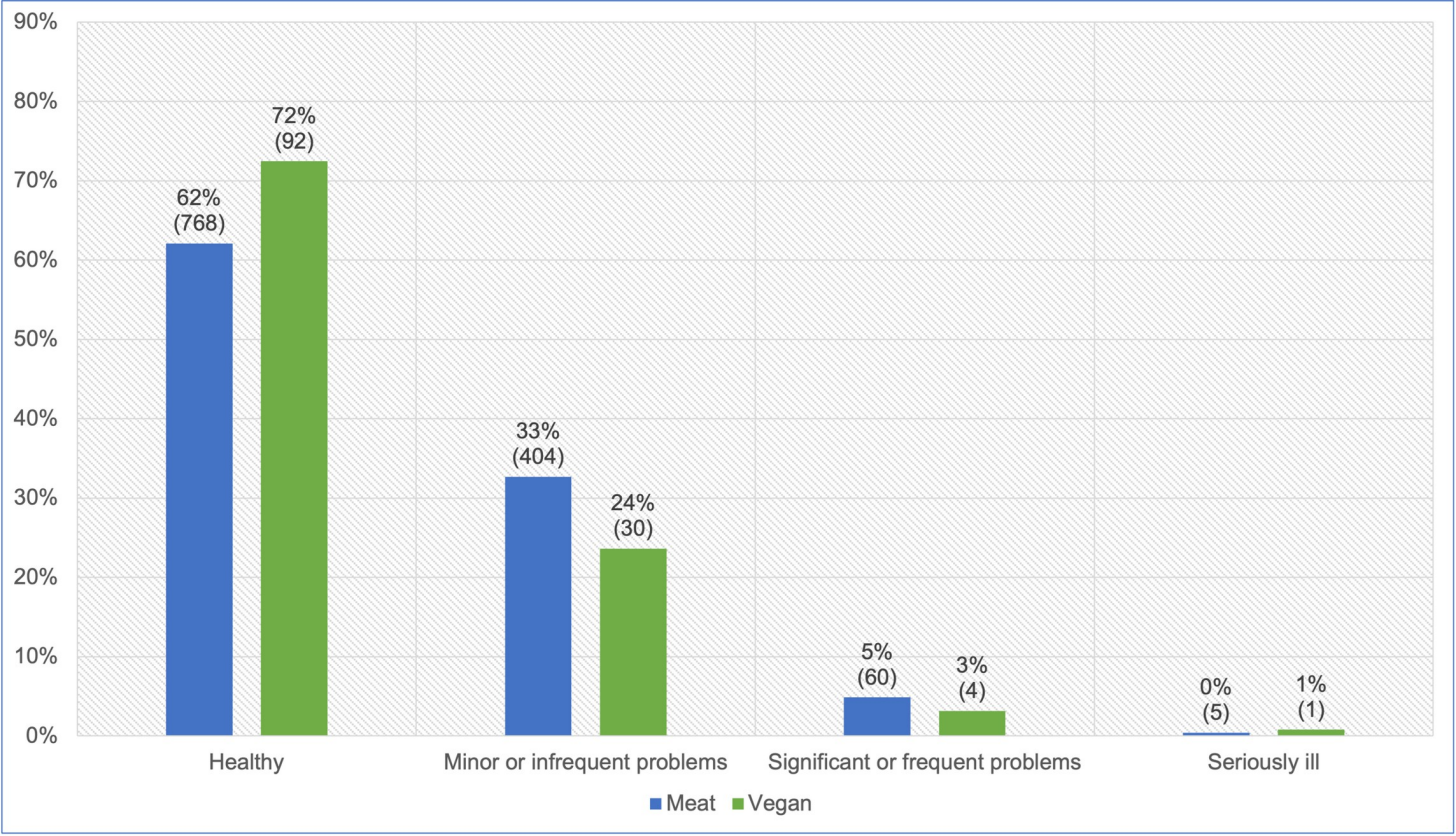
Guardian opinions of illness severity of 1,364 cats fed meat-based or vegan diets.

After excluding five ‘unsure’ responses, 1,364 guardians reported their own opinions of the illness severity of their cats (Fig 11). After controlling for cat demographic factors in the regression model, there were no significant differences in the reported guardian opinions of illness severity between cats fed meat-based and vegan diets (p = 0.1446). However, cats fed a vegan diet had, on average, 26.9% lower odds of being considered by guardians to have more severe illness, compared to cats fed meat. Because the effect was stronger than 25%, but not statistically significant, it can be considered a *strong tendency*. For an average cat, this represented a 22.8% reduction in the risk of reportedly being considered by guardians to have more severe illness.

### Specific disorders (22)

As noted, 1,004 cats saw veterinarians at least once in the last year. After excluding 11 cases in which guardians were unsure of their veterinarians’ assessments, guardians were reportedly sure of the assessments of 993 veterinarians (Fig 10). In total 577 of these cats were considered entirely healthy. The remaining 416 cats were considered to suffer from one or more disorders. In five cases (all fed conventional meat), details were not provided, or veterinarians reportedly assessed cats as ‘healthy’, ‘old’, or variations of these–i.e., not unwell, as reported. These cats were excluded. The remaining 411 cats were analysed. In 58 cases, details of ‘other’ disorders were reported. These were examined, and then reclassified into the 18 existing, or four new disorder types. In total, respondents reported that these 411 cats were considered by their veterinarians to be suffering from 652 cases of 22 specific disorders (Table 6).

**Table 6:** Prevalence of 22 specific disorders or affected bodily systems in 988 cats fed meat-based or vegan diets, based on reported assessments of veterinarians. Note: Relative frequencies state the prevalence in the respective group (meat-based, vegan, or overall). Rank order reflects overall prevalence. For each disorder, a separate additive logistic regression model was estimated to investigate dietary associations, which included age (nonlinear), sex and neuter status and primary location as linear control variables. These were used to estimate p-values. Boldface indicates p < 0.05.

Rank	Disorders (22)	Meat	Vegan	Overall	p-value
1	Dental/oral (teeth/mouth)	103 (11%)	7 (8%)	110 (11%)	0.8604
2	Body weight	79 (9%)	5 (6%)	84 (9%)	0.5557
3	Gastrointestinal (e.g., diarrhoea, vomiting)	60 (7%)	2 (2%)	62 (6%)	0.0934
4	Skin/coat	49 (5%)	3 (3%)	52 (5%)	0.3608
5	Hormonal (e.g., diabetes, hyper-/hypothyroidism, Addison’s, Cushing’s)	37 (4%)	2 (2%)	39 (4%)	0.5800
6	Lower urinary tract	31 (3%)	4 (4%)	35 (4%)	0.7661
7	Kidney	29 (3%)	4 (4%)	33 (3%)	**0.0235**
8	Heart	29 (3%)	2 (2%)	31 (3%)	0.6803
9	Eyes	23 (3%)	3 (3%)	26 (3%)	0.6534
10	Mobility	25 (3%)	0 (0%)	25 (3%)	1.0000
11	Other medical	23 (3%)	2 (2%)	25 (3%)	0.9648
12	Ears	19 (2%)	4 (4%)	23 (2%)	0.4172
13	Respiratory tract (airways/lungs)	19 (2%)	1 (1%)	20 (2%)	0.5550
14	Other musculoskeletal (muscle or bone)	16 (2%)	0 (0%)	16 (2%)	1.0000
15	Cancer/tumours	15 (2%)	0 (0%)	15 (2%)	0.9988
17	Injury	13 (1%)	0 (0%)	13 (1%)	1.0000
16	Behavioural	11 (1%)	2 (2%)	13 (1%)	0.3789
18	Anal glands	9 (1%)	0 (0%)	9 (1%)	1.0000
19	Liver	7 (1%)	1 (1%)	8 (1%)	0.2916
21	Viral e.g. FELV, FIV	5 (1%)	0 (0%)	5 (1%)	1.0000
20	Allergy	4 (0%)	1 (1%)	5 (1%)	0.4145
22	Internal parasites	3 (0%)	0 (0%)	3 (0%)	1.0000
	**Total cats: healthy and unwell**	898	90	988	

#### Proportion of unwell cats and average number of disorders per unwell cat

In addition to these 411 cats with 652 cases of 22 specific disorders, respondents reported that the remaining 577 cats were considered by their veterinarians to be healthy. Overall, 42% suffered from at least one disorder, and the average number of disorders per unwell cat was 1.59 (Table 7).

**Table 7:** 652 occurrences of 22 specific disorders in in 988 cats fed meat-based or vegan diets.

Health status	Meat	Vegan	Total
Unwell	378	33	411
Healthy	520	57	577
**Total cats**	**898**	**90**	**988**
**% unwell**	**42%**	**37%**	**42%**
Cases of disorders	609	43	652
**Average cases/unwell cat**	**1.61**	**1.30**	**1.59**

#### Reported veterinary assessment of being unwell

After controlling for cat demographic factors in the regression model, there was no statistically significant association between diet type and the reported number of unwell cats (p = 0.7836). Cats fed a vegan diet had, on average, 6.4% lower odds of being considered unwell, compared to cats fed meat. Because the effect was smaller than 10%, and not statistically significant, it can be considered *marginal*. For an average cat, this represented a 3.6% reduction in the risk of reportedly being considered unwell.

#### Number of disorders per unwell cat

The number of disorders per unwell cat ranged from one to seven (Table 8). For the 411 cats reportedly assessed by veterinarians to be suffering from a disorder, vegan cats had less disorders, on average, than those fed meat. The mean number of disorders were: meat-based = 1.61 (sd 0.985), range 1–7, and vegan diets = 1.30 (sd 0.637), range 1–3. After controlling for cat demographic factors in the regression model, there were no significant differences in the average number of health disorders between the dietary groups (p = 0.1525). However, cats fed a vegan diet had, on average, 15.5% fewer health disorder cases, compared to cats fed meat. Because the effect was stronger than 10%, but not statistically significant, it can be considered a *tendency*.

**Table 8:** Number of disorders per unwell cat, amongst 411 cats fed meat-based or vegan diets.

Disorders per unwell cat	1	2	3	4	5	6	7
Meat	231	99	26	11	9	1	1
Vegan	26	4	3	0	0	0	0

#### Prevalence of 22 specific disorders

**Fig 12:**
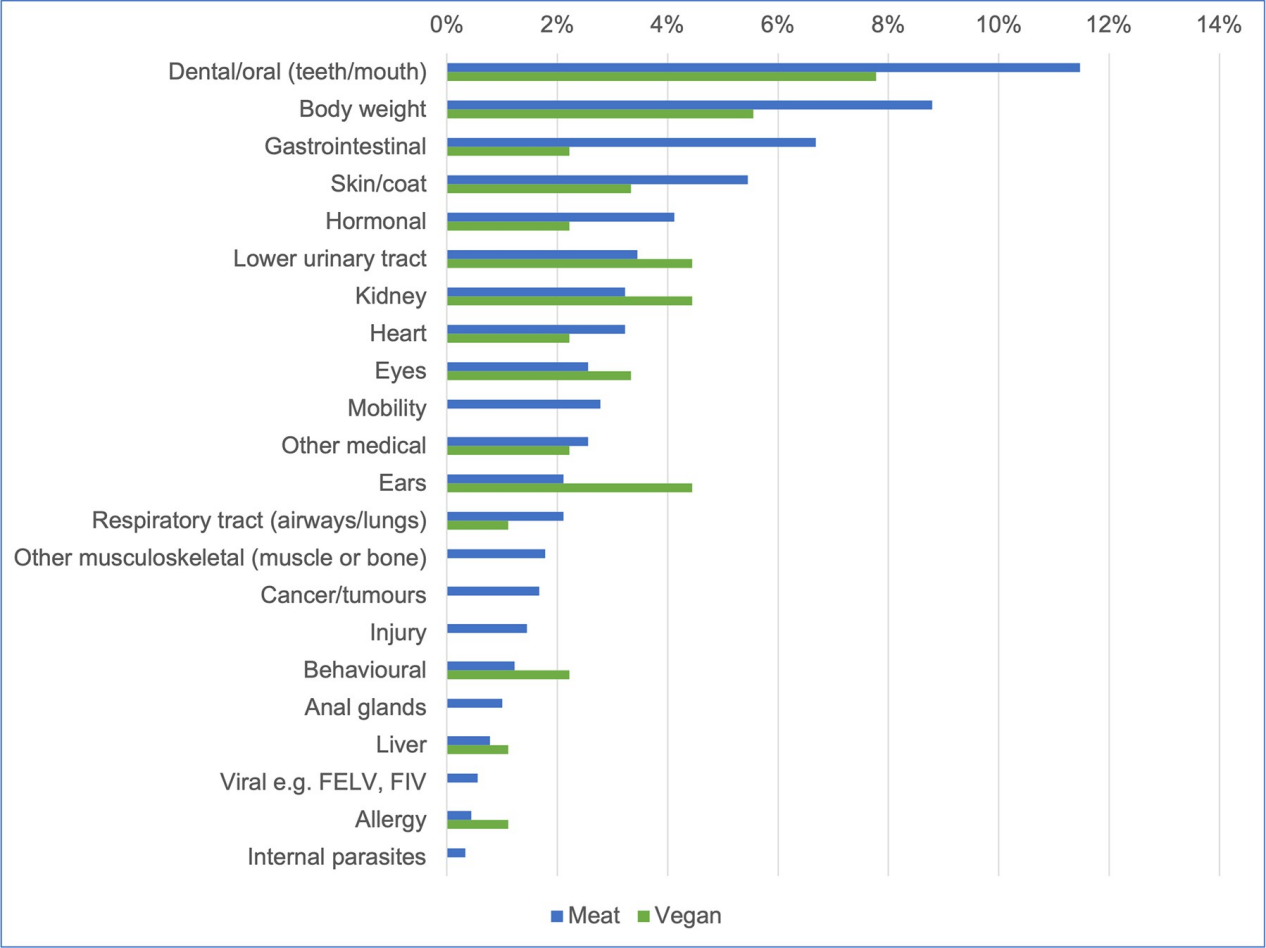
Prevalence of 22 specific disorders or affected bodily systems in 988 cats fed meat-based or vegan diets, based on reported assessments of veterinarians. Note: Vertical axis order reflects overall prevalence of disorders (combining all diets).

The prevalence of these 22 specific disorders in these 988 cats is indicated in Table 6 and Fig 12.

#### Differences between dietary groups

Based on probability of occurrence, the 10 most common disorders found within each dietary group are listed in Table 9. Probabilities of suffering from a disorder respectively appeared highest in meat-based cats (for 15 disorders) and vegan cats (for seven disorders). However, with the exception of kidney disease, differences between diet groups were not statistically significant (Table 6).

**Table 9:** The 10 most common disorders or affected bodily systems among 988 cats fed meat-based or vegan diets, based on reported assessments of veterinarians. Note: Percentages provide the prevalence of each disorder within each dietary group, and overall.

Rank	Meat	Vegan	Overall
1	Dental/oral (teeth/mouth) (11%)	Dental/oral (teeth/mouth) (8%)	Dental/oral (teeth/mouth) (11%)
2	Body weight (9%)	Body weight (6%)	Body weight (9%)
3	Gastrointestinal (e.g., diarrhoea, vomiting) (7%)	Lower urinary tract (4%)	Gastrointestinal (e.g., diarrhoea, vomiting) (6%)
4	Skin/coat (5%)	Kidney (4%)	Skin/coat (5%)
5	Hormonal (e.g., diabetes, hyper-/hypothyroidism, Addison’s, Cushing’s) (4%)	Ears (4%)	Hormonal (e.g., diabetes, hyper-/hypothyroidism, Addison’s, Cushing’s) (4%)
6	Lower urinary tract (3%)	Skin/coat (3%)	Lower urinary tract (4%)
7	Kidney (3%)	Eyes (3%)	Kidney (3%)
8	Heart (3%)	Gastrointestinal (e.g., diarrhoea, vomiting) (2%)	Heart (3%)
9	Mobility (3%)	Hormonal (e.g., diabetes, hyper-/hypothyroidism, Addison’s, Cushing’s) (2%)	Eyes (3%)
10	Eyes (3%)	Heart (2%)	Mobility (3%)

For several disorders, guardians had the option to provide additional information. With respect to dental/oral problems, further information was provided by all 110 respondents. The most common causes, in order, were dental calculus/plaque/tartar, gingivitis, and a variety of ‘other’ causes. With respect to body weight problems, 83 of 84 respondents described whether cats were over- or underweight. Sixty six percent (55) of the 83 reported cases were overweight, and 34% (28) were underweight. With respect to skin/coat problems, further information was provided by all 52 respondents. The most common causes, in order, were atopic/allergic dermatitis (inflamed skin due to allergies), hair loss (unspecified), and a variety of ‘other’ causes. With respect to eye problems, further information was provided by all 26 respondents. The most common causes were infections (unspecified). Also common were feline herpes virus, conjunctivitis, and ’irritated’ eyes. With respect to mobility problems, further information was provided by all 25 respondents. The most common causes, in order, were osteoarthritis/arthritis, and a variety of ’other’ causes.

## Discussion

### Importance of health to guardians

Our results affirmed the importance of pet health to guardians. Among 1,380 respondents who played some role in choosing cat diets, health and nutrition was the factor considered most important in purchasing decisions. It was cited as important by 85% of respondents, and ‘maintenance of pet health’ was the most important sub-factor cited. Given that 90% of respondents overall did not work in the pet or veterinary industries, this probably closely matches the level of concern in the general population. These results concur with those of other studies. ‘Health & Nutrition’ was the most important among 24 pet food characteristics ranked by 2,181 pet guardians, of whom 74% were not animal professionals, in a US-based study from 2015–2016 by Schleicher *et al*. [47].

Of our respondents feeding conventional or raw meat-based diets, 51% stated they would realistically consider alternatives. These results were slightly higher than those of Dodd *et al*. [48], who surveyed 3,673 primarily Canadian and US cat or dog guardians. They found that 35% (1,083/3,130) of responding guardians who did not yet feed a plant-based diet to their pet, were interested in doing so. We found that the most important attribute such alterative diets would need to provide, was ‘Confidence about pet health’ (considered necessary by 83% of respondents). Dodd *et al*. [48] found that the most important attributes such an alternative diet would need to provide, were ‘further evidence of nutritional sufficiency’ (45% - 269/599), followed by veterinary approval (20% - 122/599), and greater availability (20% - 117/599).

### Feline demographics

Health status may be affected by age, sex and desexing (neutering) status [34, 35]. Hence, we sought to ascertain differences between major dietary groups in these characteristics, and to compare our sample averages with those of normal cats.

Both dietary groups we studied appeared to have an age distribution broadly representative of normal cats (Fig 4), with a median age of eight years overall. In comparison, among 906 primarily North American cats, Dodd *et al*. [17, pp. 2–3] reported a mean cat age of 7.5 years (std. dev. 4.85), and no association between age and diet. In a study of 8,175 Finnish cats, Vapalahti *et al*. [49] found a mean age of 5.4 years. At 6.24 years, the average age of cats in our sample fed vegan diets was nearly two years less than that of those fed meat-based diets, at 8.14 years, and the difference was significant. This was noteworthy, as younger cats may have decreased risks of certain health disorders. However, our regression models controlled for the possible effects of age and other feline demographic factors, when considering the effects of diet on health outcomes.

The sex/neuter status within our sample also appeared broadly representative of normal cats, with just above half (52%) being female. Similarly, Dodd *et al*. [17] found 52% of cats they studied were female, and Vapalahti *et al*. [49] found 53% of their studied cats were female. Around 97% of cats we studied were neutered, with neutering nearly as common in females, as males, and between diet groups (Fig 5). Dodd *et al*. [17] similarly found 97% of cats they studied were neutered. In comparison, Vapalahti *et al*. [49] found 72% of cats in their sample to be neutered.

### General indicators of illness (7)

#### Increased veterinary visits

We considered seven general indicators of illness, of which the first was the number of veterinary visits in the previous year. Routine health checks and administration of preventative healthcare, such as vaccinations, are normally conducted annually [50–52]–at least in the UK, where 70% of survey respondents were based. Visit numbers may increase somewhat for kittens or geriatric animals, but these comprised a low proportion of studied animals (Fig 4). Hence, zero or one veterinary visits in the previous year would normally be consistent with good health, for our sample. In contrast, two, three or more visits could indicate a health concern. Cats fed meat-based diets appeared slightly more likely to fall within the latter group than those fed vegan diets (31% vs 27% respectively) (Fig 7). After controlling for feline demographic factors via our regression model, it was apparent that cats fed a vegan diet had, on average, 10.3% lower odds of having two or more veterinary visits. Although not statistically significant, this indicated a *tendency* toward this indicator of better health.

#### Medication use

Medication use was similarly considered to indicate a probable health concern, and was indicated by 31% of guardians feeding meat-based diets, compared with 24% of those feeding vegan diets (Fig 8). After controlling for feline demographic factors via our regression model, it was apparent that cats fed a vegan diet had, on average, 19.6% lower odds of receiving medication. Although not statistically significant, this indicated a *tendency* toward this indicator of better health.

#### Progression onto a therapeutic diet

Guardians were also asked whether their cat progressed onto a therapeutic diet, after being fed primarily a meat-based or vegan diet for at least one year. Such progression was similarly considered indicative of a possible health concern. Affirmative answers were provided by 8% of those feeding meat-based diets, compared with 4% of those feeing vegan diets (Fig 9). After controlling for feline demographic factors via our regression model, it was apparent that cats fed a vegan diet had, on average, 56.5% lower odds of progressing onto a therapeutic diet. Although not statistically significant, this indicated a *strong tendency* toward this indicator of superior health. However, this particular health indicator may have been affected by a reluctance of guardians feeding vegan diets, to progress their cats onto therapeutic diets, if these were not also vegan. By 2023, few feline therapeutic diets were vegan. In contrast, meat-based therapeutic diets were readily available to guardians choosing to feed meat-based diets.

#### Reported veterinary assessment of being unwell

After limiting to cats who had seen a veterinarian at least once in the previous year, and excluding cats for whom guardians were unsure of their veterinarians’ assessments, and five cases in which details were not provided or veterinarians reportedly did not consider cats to be truly unwell, 988 cats remained (Table 7). Forty-two percent of these cats were considered to suffer from at least one health disorder. Similar results were found by Dodd *et al*. [17] who found 48% of 1,208 primarily North American cats suffered from at least one disorder. In our sample, the percentage of unwell cats was slightly higher for cats fed meat (42%) than for those fed vegan diets (37%). After controlling for feline demographic factors via our regression model, it was apparent that cats fed a vegan diet had, on average, 6.4% lower odds of being considered unwell. Although not statistically significant, this indicated a *marginal tendency* toward this indicator of better health.

#### Reported veterinary assessment of more severe illness

Significant, frequent or serious healthcare problems were indicated for 10% and 7% of cats fed meat-based and vegan diets respectively (Fig 10). After controlling for feline demographic factors via our regression model, it was apparent that cats fed a vegan diet had, on average, 9.7% lower odds of reportedly being assessed by veterinarians as having more severe illness. Although not statistically significant, this indicated a *tendency* toward this indicator of better health.

#### Guardian opinion of more severe illness

A similar pattern was revealed when guardians were asked for their own opinions of their cats’ health status–albeit with a shift of roughly 5–10% in all groups, toward viewing cats as healthier than veterinarians were expected to rate them. Guardians assessed animals as suffering from healthcare problems, for 38% of cats fed meat-based diets, and 28% of those fed vegan diets. Significant, frequent or serious healthcare problems were indicated for 5% and 4% of cats fed meat-based and vegan diets respectively (Fig 11). After controlling for feline demographic factors via our regression model, it was apparent that cats fed a vegan diet had, on average, 26.9% lower odds of being considered by guardians to have more severe illness. Although not statistically significant, this indicated a *strong tendency* toward this indicator of better health.

#### Consistency of guardian opinions with reported veterinary assessments

When comparing guardian’s own opinions of their cat’s health status with the reported assessments of their veterinarians, 74.9% of guardians agreed with reported veterinary assessments. However, 12.8% of guardians felt their cat was healthier, and 12.3% felt their cat was less healthy than the reported assessments of their veterinarians.

#### Number of disorders per unwell cat

Within our sample, cats considered unwell were reported to suffer from a greater number of medical disorders when fed meat, than when fed vegan diets, suffering from an average of 1.61 and 1.30 disorder respectively (Table 7). After controlling for feline demographic factors via our regression model, it was apparent that cats fed a vegan diet had, on average, 15.5% fewer health disorder cases. Although not statistically significant, this indicated a *tendency* toward this indicator of better health.

#### General illness indicators overall

Jointly considering these seven general indicators of illness, the cats within our sample who were fed vegan diets appeared healthier than those fed meat-based diets. They had better health outcomes for every indicator studied. The cats fed vegan diets were younger on average, which can be health protective. Yet, even after controlling for the effects of age, sex, neutering status and primary location via regression models, there remained a *strong tendency* toward better health outcomes for cats fed vegan diets in two cases, a *tendency* in four cases, and a *marginal effect* in one case (Table 4). The ‘strong tendency’ effect may have been inflated in the case of progression on to therapeutic diets, by limited availability of vegan therapeutic diets. Furthermore, none of these effects reached the level of statistical significance in the samples studied. The limited numbers of cats within our sample who were fed vegan diets (127 of 1,369 studied, or 9.3%) may have prevented the detection of statistically significant effects to some extent. Nevertheless, in accordance with state-of-the-art statistical practice and the American Statistical Association’s statement on the use of p-values [43, 44], our interpretations of the importance of apparent effects were based mainly on quantification of their magnitudes, rather than on p-values. Accordingly, when jointly considering these seven general indicators of illness, the overall trend was clear and consistent: cats fed vegan diets had better health outcomes than those fed meat. For average cats, relative reductions in occurrences associated with vegan diets mostly ranged from ~7% to ~23% (Table 5).

These results appear to broadly concur with those of previous studies. Dodd *et al*.’s 2021 study [17] noted that in most respects the health of vegan cats was reportedly similar to that of those fed meat-based diets, without statistically significant differences. However, in some respects vegans appeared to enjoy advantages: more guardians of vegan cats, than of those fed meat-based diets, reported their cat to be in very good health. Dodd *et al*. included 1,026 cats whose diets were known, of whom 187 (18%) were fed vegan diets.

Semp’s 2014 study [19] of vegan cats and dogs included 15 vegan cats. During standardized clinical examinations, no abnormalities were detected that appeared associated with the vegan diet. Wakefield *et al*.’s 2006 study [25] of 52 cats maintained on conventional diets, and 34 on vegetarian diets, found no significant differences in perceived health status, with most caregivers in both groups describing their cats as healthy or generally healthy. In Kienzle and Engelhard’s 2001 study [26] of eight vegetarian cats, three cats displayed symptoms of health problems. Leon *et al*.’s 1992 study [27] also found health problems in cats maintained on vegetarian diets. However, in both of these studies the diets had significant nutritional deficiencies, limiting their relevance for cats maintained on vegan diets formulated to be nutritionally sound, as modern commercial diets generally are [14].

### Specific disorders (22)

The ten most common disorders overall (i.e., regardless of diet) within these 988 cats were reportedly: dental/oral (11%), body weight (9%), gastrointestinal (e.g., diarrhoea, vomiting) (6%), skin/coat (5%), hormonal (e.g., diabetes, hyper-/hypothyroidism, Addison’s, Cushing’s) and lower urinary tract (both 4%), kidney, heart, eyes and mobility (all 3%) (Table 9, Fig 12).

After collecting data for 1,208 North American cats, Dodd *et al*. [17] found the 10 most common reported health disorders (with prevalences) to be dental disease (17%), dermatopathy (skin) (11%), lower urinary tract disease (11%), gastrointestinal and hepatic diseases (10%), obesity (8%), ocular disorders (5%), endocrinopathy (hormonal) (3.2%), renal disease (3%), cardiac disease (2.2%), and neoplasia (1.7%). This study reported the mean durations cats had been fed their diets as 3.6 years for those fed vegan diets, and 3.8 years for all cats.

In their study of study of 8,175 Finnish cats, Vapalahti *et al*. [49] found the 10 most common disease categories overall (with prevalences) to be dental and oral diseases (28%), genitals of female cats (17%), skin (12%), the urinary system (12%), parasites and protozoans (11%), the digestive tract (11%), eyes (10%), the musculoskeletal system (10%), behaviour (9%) and respiratory tract (8%).

In their study of health conditions reported by 469 US cat guardians, Freeman *et al*. [33] found the 10 most common disease categories overall (with prevalences) to be dental disease (2.3%), lower urinary tract disease (2.1%), gastrointestinal tract or hepatic disease (1.7%), diabetes mellitus (1.5%), unspecified (1.5%), musculoskeletal disease, cardiac disease, infectious disease, kidney (all 1.1%), and other endocrine (hormonal) disease (0.9%).

Within our sample, probabilities of suffering from a disorder respectively appeared highest in meat-based cats (for 15 disorders) and vegan cats (for seven disorders) (Fig 12). In one case (kidney disease), cats fed vegan diets appeared slightly more likely to suffer from this disorder (4% vs. 3% prevalence), and this difference was statistically significant (p = 0.0235, Table 6). Most feline kidney disease is chronic rather than acute, and much is caused by genetic predisposition, although a range of other causes are possible [36]. In this case, it’s important to note that kidney disease was reported in only four cats fed vegan diets. Hence, this result is highly susceptible to any anomaly or misdiagnosis affecting individuals. For example, due to expected low numbers in breed groups, we did not attempt to control for effects of breed. It’s possible that breeds predisposed to kidney disease–such as Abyssinian or Persian cats–may have been more common within this sample. If just one of these cats had not suffered from kidney disease after all (e.g., after any breed effects were controlled for), the prevalence would have changed to 3% (equivalent to those of the meat-based cats), and this result would no longer have been statistically significant (p-value = 0.0899).

In comparison, Dodd *et al*. [17] studied the prevalence of kidney (and other) diseases, in 1,208 cats on varied diets. The overall prevalence of kidney disease was 3%. Among 667 meat-based cats this was 4.7%, and amongst a total of 256 cats fed a vegan diet this was 1.2% (three in total). This difference was not statistically significant, after using regression models to control for age, breed type, sex, and body condition score. These 256 cats included some whose diets may have been supplemented by animal-derived treats/snacks/supplements and/or outdoor hunting–which may also have been true of some of the cats we studied. In one of the largest such studies, O’Neill *et al*. [53] examined the prevalence of multiple disorders in 3,584 English cats based on veterinary clinical records from 1 September 2009 to 15 January 2014. Kidney disease was present in 149 cats overall (4.2%; 95% CI 3.5–4.8), a result consistent with our finding of 4% for cats fed vegan diets. Hence, caution must be exercised to avoid overinterpreting the significance of apparent differences based on only four cats fed vegan diets, as observed in our study sample.

For all other health disorders, differences between dietary groups were not statistically significant, and in most cases sample sizes were similarly small (Table 6). The largest such group comprised only seven vegan cats, who suffered from dental/oral disorders. Despite the limited generalisability of small numbers, results within our sample were nevertheless interesting in some cases. The cats in our sample fed vegan diets appeared to have dramatically lowered rates of mobility problems, other musculoskeletal disease, cancer/tumours, injuries, anal gland problems, viral problems e.g., FELV, FIV, and internal parasites. They also appeared to have around half or lower rates of gastrointestinal, skin/coat, hormonal or respiratory tract problems. On the other hand, cats fed meat-based diets appeared to have substantially lowered rates of ear problems, behavioural problems and allergies.

Some of these results match current understanding that some of these disorders may be related. Musculoskeletal disease may cause mobility problems [28, p. 783]. The latter are also more common in cats who are overweight. Within this study sample the proportion suffering from body weight disorders were recorded as 9% and 6% of cats fed meat-based and vegan diets respectively. In total 66% of cats with body weight disorders were reportedly overweight. Cats who roam outdoors are more likely to fight with other cats, and to suffer injuries, and to acquire viruses such as FELV, FIV, and parasites [28, p. 500]. Within this study sample, cats who mostly stayed indoors, comprised 57% and 67% of cats fed meat-based and vegan diets respectively. Some have suggested that keeping cats inside may also increase the frequency of behavioural problems [54], which appeared more common in the cats fed vegan diets. In other cases, no immediately obvious aetiological explanation is available, such as the apparently increased risks of ear problems and allergies reported among cats fed vegan diets.

The apparently decreased rates of some specific disorders among the vegan cats in our sample, concur with some other results. Dodd *et al*.*’s* 2021 study [17] included 1,026 cats whose diets were known, of whom 187 (18%) were vegan. Fewer of the latter were reported to have gastrointestinal and hepatic disorders, or body weight problems, compared to cats fed meat-based diets. In our sample of 988 cats of whom 90 were vegan (Table 7), those fed vegan diets appeared to have dramatically lowered rates of gastrointestinal problems, and lowered rates of body weight problems. Slightly higher rates of liver problems were reported, but with only eight cats in total, including one vegan, suffering from a liver problem, differences between these dietary groups were not statistically significant (Table 6).

Semp’s questionnaire to 174 vegan dog and 59 vegan cat guardians resulted in 38 reports of healthier and shinier coats after transitioning to vegan diets, and 16 guardians described improved odours of their pets. Some dermatological problems reportedly resolved. Among 15 cats subjected to clinical examination, none had pruritic (itchy) skin other than one cat suffering from flea allergy dermatitis [19]. Within our study sample, the probabilities of a suffering from a skin/coat condition were reportedly 5% in cats fed meat-based, and 3% in those fed vegan diets, respectively (Table 6).

Some of Semp’s respondents also noted improvement of stool consistency. Within our study sample probabilities of a suffering from a gastrointestinal problem (which may affect stool consistency) were 7% in cats fed meat-based, and 2% in those fed vegan diets, respectively (Table 6).

Our results differed, however, from those of two older studies. During a study of eight vegetarian cats (and 86 vegetarian dogs), Kienzle and Engelhard reported in 2001 [26] that one cat showed symptoms of retinal atrophy, and two displayed reduced frequency of oestrus. In our sample the probabilities of eye and hormonal problems were, respectively: meat-based– 3% and 4%, vegan– 3% and 2% (Table 6). However, Kienzle and Engelhard also found multiple nutritional deficiencies in the diets fed. By the time of our survey around two decades later, vegan pet food formulations and manufacturing processes can be expected to have improved significantly.

Similarly, in 1992 Leon and colleagues [27] reported that cats maintained on a vegetarian diet had impaired neuromuscular function. This could affect mobility. In our sample the probabilities of mobility problems were respectively: meat-based– 3%, vegan– 0% (Table 6). However, the vegetarian diet fed by Leon *et al*. was formulated to be deficient in potassium, which is known to impair neuromuscular function [28, pp. 712–713].

### Study limitations

As noted in our prior study [30] utilising a similar methodology, our study had multiple limitations. To start with, our respondents were not fully representative of the cat-owning population. Those who lacked easy internet access would have been less likely or unable to complete this internet-based survey. And although most ages were well represented, men were not, representing only 9% of respondents. Most of our participants were located in the UK (70%) or Europe (20%). Hence, our results may most accurately represent female cat guardians based within the UK or Europe. However, we see no reason why these anomalies would have significantly affected reported opinions and data concerning animals.

When reporting diets fed, guardians were asked to “consider the main ingredients within your pet’s normal diet”. Diets were then assessed as meat-based or vegan. Given our large respondent numbers, many variations of these diets would have been used. Our survey did not inquire about nutritional formulation, such as compliance with FEDIAF or AAFCO nutritional guidelines, which is important to ensure diets are complete and balanced with respect to all essential nutrients and elements.

Furthermore, these diets were not fed exclusively. Of the 1,369 cats within the two main diet groups, 41% received a variety of treats at least once daily, and 13% were also regularly offered dietary supplements. Additionally, 42% of cats overall inhabited a mixed or mostly outdoor habitat. For those fed vegan diets, these were 29% (mixed) and 4% (mostly outdoor) (Table 1). It is possible that some cats, especially those in the latter groups, may have supplemented their diets by hunting. Accordingly, it is important to note that our results indicate health outcomes when cats are fed these diet types within normal households, with normal feeding regimes, rather than when cats are exclusively fed each of the two main diet types, as might occur within a controlled study within a research institute.

Additionally, our study relied on quantitative information and opinions provided by guardians. The most reliable medical studies are large-scale, prospective studies, that ideally utilise objective assessments of unambiguous data. Veterinary clinical examinations, and veterinary assessments of animal health status, may normally be expected to be more reliable than guardian opinions, and laboratory results of physiological parameters such as blood and urine tests can provide particularly objective data. However, when large animal numbers are involved, as is necessary for statistical validity of results, such studies become expensive. Unfortunately, such studies were well beyond our research budget.

Accordingly, we were forced to rely on other indicators of illness. One of these was the answers of guardians (90% of whom were not in the veterinary or pet industries), about health indicators relating to their cats. We acknowledge that reliance on guardians limits the reliability of our results, for example, due to lapses in memory. Our guardians most at risk of this, were those 7.8% (107/1,369) whose animals subsequently progressed onto a therapeutic diet, after initial maintenance on meat-based or vegan diets (Fig 9). These guardians were asked to “answer all questions about your animal and their diet, using the 12 months prior to starting their therapeutic or prescription (i.e., medical) diet.” Hence, these guardians were asked to recall details more historical in nature. However, these key instructions were highlighted within the survey, and respondents were also instructed, “If you cannot recall details, please provide your best estimates, or answer ’unsure’ etc. as appropriate”.

Another source of potential error, when relying on guardian answers, is unconscious bias. This could occur if a guardian using a conventional or unconventional pet diet expected a better health outcome as a result, and if this expectation exerted an unconscious effect on their answers about pet health indicators. Our study included more vegans than reported in some other studies [55]. It is conceivable that vegans, or respondents following other dietary groups, such as omnivores, might have had greater subconscious expectations of good health, when animals were fed diets similar to their own. We acknowledge such possible unconscious bias effects cannot be fully eliminated, but to minimise their effects on reported results, we ensured that survey questions asking about animal health were positioned prior to questions about animal diets. This minimises chances that answers might be affected by prior answers about dietary choices, e.g., if a guardian reporting use of an unconventional diet, subsequently became more likely to consciously or unconsciously under-report health problems. Additionally, by careful wording choice, no bias for or against any particular diet was implied within survey advertising materials, or within the survey questions or explanatory text. We do not consider that any remaining unconscious bias effects would be appreciably greater in one dietary group than another, and hence consider that their effects on our results were probably minimal, overall.

Despite such steps, reliance on guardian-reported answers is vulnerable to error. We sought to minimise the impact of this unavoidable limitation, by also asking guardians to additionally report the assessment of their veterinarians, concerning their animals’ health. To increase the reliability of such reported veterinary assessments, we further analysed only those responses from guardians whose animals had seen a veterinarian at least once in the previous year, and who were certain of their veterinarian’s assessment. Responses from those who were uncertain, were excluded. And as mentioned, guardians were also given the opportunity to report their own opinion. It was expected the knowledge they would be able to provide their own opinion, if they disagreed with their veterinarian, would encourage them to more accurately report the assessments of their veterinarian. However, we ensured that the analysis of specific health disorders relied on reported veterinary assessments alone, rather than on guardian opinions.

We also asked about several more objective general indicators of illness, including the frequency of veterinary visits, and the use of any medications, within the last year, as well as progression onto a therapeutic diet, after being fed a meat-based or vegan diet for at least one year. Whilst we accept that a small number of these reported data and assessments may have been incorrect, we do not consider it plausible that a significant proportion of them were incorrect.

Our survey was made available from May–December 2020, during the global coronavirus (COVID-19) pandemic. Subsequent lockdowns may have decreased the frequency of veterinary visits in some regions, and potentially, the use of medications or therapeutic diets prescribed by veterinarians. For example, 70% of respondents stated they were from the UK, and in 2020, UK lockdowns occurred during all or part of March, April, July, and September to December [56]. The implementation of remote veterinary consultations and prescribing in many regions may have partly mitigated this effect. Nevertheless, we acknowledge this may have lowered the frequency of some health indicators such as the number of veterinary visits, and medication or therapeutic diet use, to a degree. However, because these were generally indicative of a possible health problem, decreased rates of these would have made our results more conservative overall. Additionally, we know of no reason why any dietary group would be more affected than another, in these respects.

Finally, although our participant numbers were sufficient to draw conclusions concerning the overall health of cats maintained on the two main diets studied, numbers affected by certain medical disorders may have been insufficient to detect statistically significant differences in risks between diet groups.

### Recommendations for safeguarding health

Within this sample of 1,369 cats, the reported data and opinions of guardians demonstrated that cats fed vegan diets appeared healthier overall, than cats fed meat-based diets. However, all dietary choices may include some hazards. Those feeding unconventional diets should take special care to ensure their diets are nutritionally complete and reasonably balanced, and appropriate for life stage (e.g., young, old) and physiological status (e.g., pregnant, heavily exercising). Several studies of vegan or vegetarian diets [17, 18, 57, 58], as well as meat-based diets [59, 60], have demonstrated that some diets in all groups have been previously formulated with nutritional deficiencies. Consumers should be encouraged to check labelling claims of nutritional adequacy, and to ask manufacturers what steps they take, and what evidence they can provide, to ensure nutritional soundness and consistency of their diets [18, 61].

Concerns have previously been voiced that vegan cats may experience urinary alkalinisation, predisposing to urolithiasis and lower urinary tract dysfunction [18]. However, neither our study (Table 6), nor Dodd *et al*.’s 2021 study [17], showed any increased risk of lower urinary tract dysfunction in vegan cats. Dodd *et al*. studied 1,026 cats whose diets were known, of whom 187 (18%) were fed vegan diets. We studied 1,369 cats, of whom 127 (9%) were fed vegan diets. Hence, this concern appears to have been unfounded.

### Suggestions for further research

As we’ve noted previously [30], large-scale cross-sectional or ideally, longitudinal studies of cats maintained on different diets, utilising objective data, such as results of veterinary clinical examinations and laboratory data, as well as veterinary medical histories, should yield results of greater reliability, if sufficient funding could be sourced.

Whether utilising such an improved research design, or an internet survey, significantly larger numbers might allow detection of statistically significant differences in risks of specific veterinary medical disorders, between dietary groups. Health consequences of other dietary groups, such as vegetarian animals, and of new diets as these become available, could also be investigated.

## Conclusions

Vegan pet foods are among a range of alternative diets being formulated to address increasing concerns of consumers about traditional pet foods, such as their ecological ‘pawprint’, perceived lack of ‘naturalness’, health concerns, or impacts on ‘food’ animals used to formulate such diets [8, 9, 48]. Critics have asserted, albeit without evidence, that vegan diets are nutritionally unsound for cats, and that guardians who feed such diets to cats may be committing animal welfare offences [12, 13].

By 2020 no very large-scale study of cats had been published, comparing health indicators between cats maintained on vegan or meat-based diets. Previous studies in this field included relatively small numbers of cats (e.g., Kienzle and Engelhard [26], n = 8 vegetarian cats; Wakefield *et al*. [25], n = 34 vegetarian cats; Semp [19], n = 174 surveyed guardians, with clinical examination and blood tests on 15 cats). In 2021, Dodd *et al*. [17] published the first very large-scale study, including 1,026 cats whose diets were known. The 187 (18%) cats fed vegan diets reportedly enjoyed health as good, and in some respects better, than those fed meat-based diets.

Our study included 1,418 cats and their guardians. Among 1,380 respondents who played some role in pet diet decision-making, pet health was cited as the most important factor when choosing diets. We analysed data for 1,369 cats, of whom 127 (9%) were fed vegan diets, with the remainder fed meat-based diets. Jointly considering seven general indicators of health and 22 specific health disorders, cats fed vegan diets tended to be healthier than those fed meat-based diets. This overall trend was clear and consistent. In most cases differences between dietary groups were not statistically significant. However, small numbers of vegan cats affected by disorders may have prevented the detection of statistically significant differences between diet groups, to some extent.

The pooled evidence to date from our study, and from others in this field, indicate that cats fed nutritionally sound vegan diets may be healthier overall, than those fed meat-based diets. Regardless of diet type, diets should always be formulated to be nutritionally complete and balanced, without which adverse clinical signs may eventually be expected to occur.

## Supporting information

S1 Fig Visualization of all control variables effect estimates, on a logarithmic y-axis, for all seven regression models, among 1,369 cats fed meat-based or vegan diets, as listed in Table 3. Note: Cat numbers in some groups were lower than 1,369, as described under Results. Dots mark the exponentiated effects, with bars corresponding to 95% confidence intervals. As noted in Table 3, the reference categories for variables ‘sex and neuter’ and ‘habitat’ were ‘female, spayed’ and ‘mostly indoor habitat’, respectively. The effects of the other categories indicate average differences compared to these reference categories. For models with a nonlinear age effect the respective panel is left blank. These nonlinear age effects are visualized in S2–S6 Figs.
(TIF)

S2 Fig Exponentiated nonlinear age effect for the ‘increased veterinary visits’ model, on a logarithmic y-axis, in 1,365 cats fed meat-based or vegan diets. Note: The solid line marks the exponentiated effect estimate, and the grey interval indicates exponentiated 95% point-wise confidence intervals.
(TIF)

S3 Fig Exponentiated nonlinear age effect for the ‘medication use’ model, on a logarithmic y-axis, in 1,369 cats fed meat-based or vegan diets. Note: The solid line marks the exponentiated effect estimate, and the grey interval indicates exponentiated 95% point-wise confidence intervals.
(TIF)

S4 Fig Exponentiated nonlinear age effect for the ‘greater illness (veterinary assessment)’ model, on a logarithmic y-axis, in 993 cats fed meat-based or vegan diets. Note: The solid line marks the exponentiated effect estimate, and the grey interval indicates exponentiated 95% point-wise confidence intervals.
(TIF)

S5 Fig Exponentiated nonlinear age effect for the ‘greater illness (guardian opinion)’ model, on a logarithmic y-axis, in 1,364 cats fed meat-based or vegan diets. Note: The solid line marks the exponentiated effect estimate, and the grey interval indicates exponentiated 95% point-wise confidence intervals.
(TIF)

S6 Fig Exponentiated nonlinear age effect for the ‘number of health disorders per unwell cat’ model, on a logarithmic y-axis, in 988 cats fed meat-based or vegan diets. Note: The solid line marks the exponentiated effect estimate, and the grey interval indicates exponentiated 95% point-wise confidence intervals.
(TIF)
