# Supplementary figures and images for: Vegan versus meat-based cat food: Guardian-reported health outcomes in 1,369 cats, after controlling for feline demographic factors
Source: PLoS One. 2023 Sep 13;18(9):e0284132. doi: 10.1371/journal.pone.0284132 (PMC10499249; doi:10.1371/journal.pone.0284132)

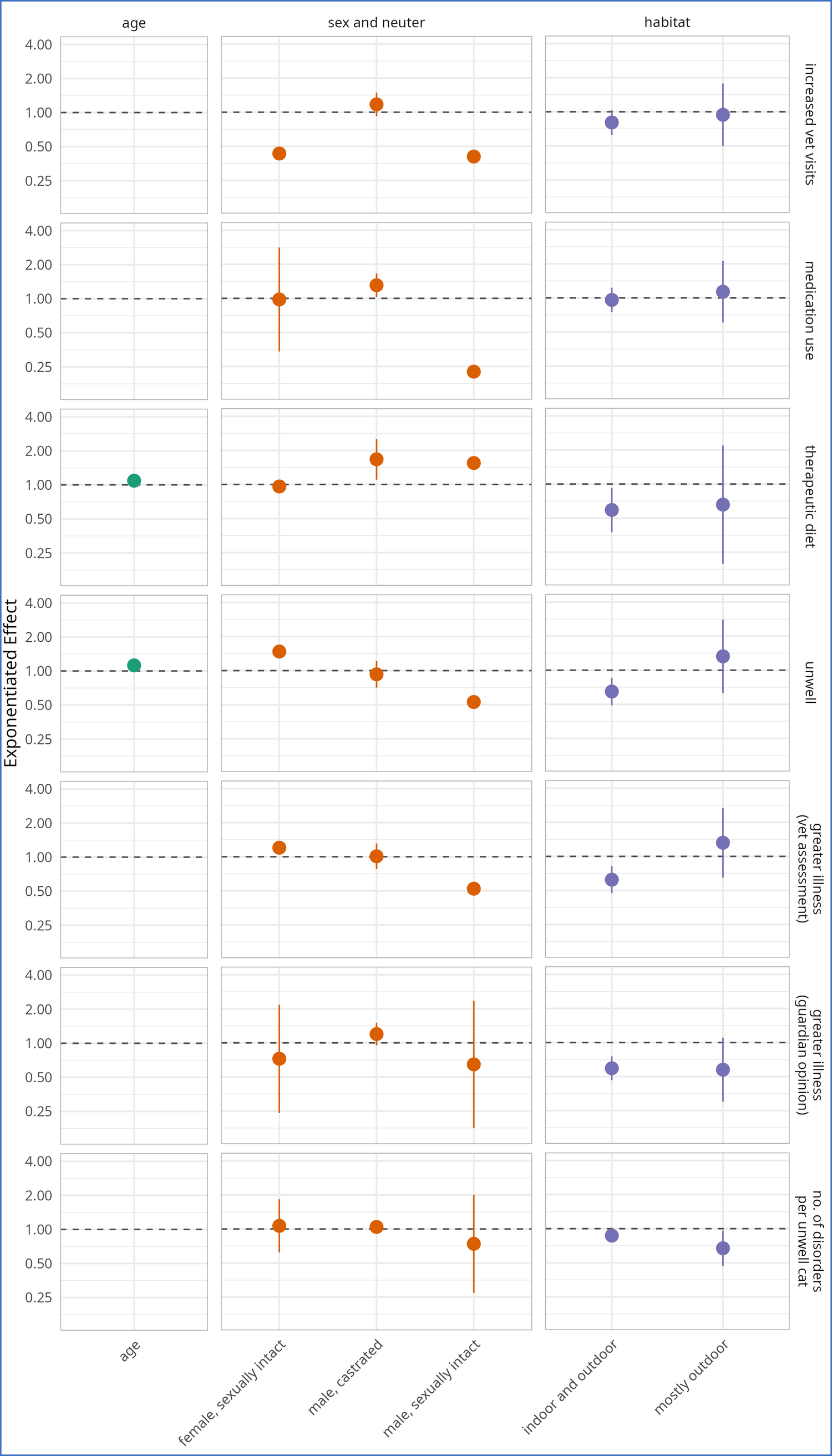

Supplement: S1 Fig — Note: Cat numbers in some groups were lower than 1,369, as described under Results. Dots mark the exponentiated effects, with bars corresponding to 95% confidence intervals. As noted in Table 3, the reference categories for variables ‘sex and neuter’ and ‘habitat’ were ‘female, spayed’ and ‘mostly indoor habitat’, respectively. The effects of the other categories indicate average differences compared to these reference categories. For models with a nonlinear age effect the respective panel is left blank. These nonlinear age effects are visualized in S2–S6 Figs. (TIF) [file pone.0284132.s001.tif]

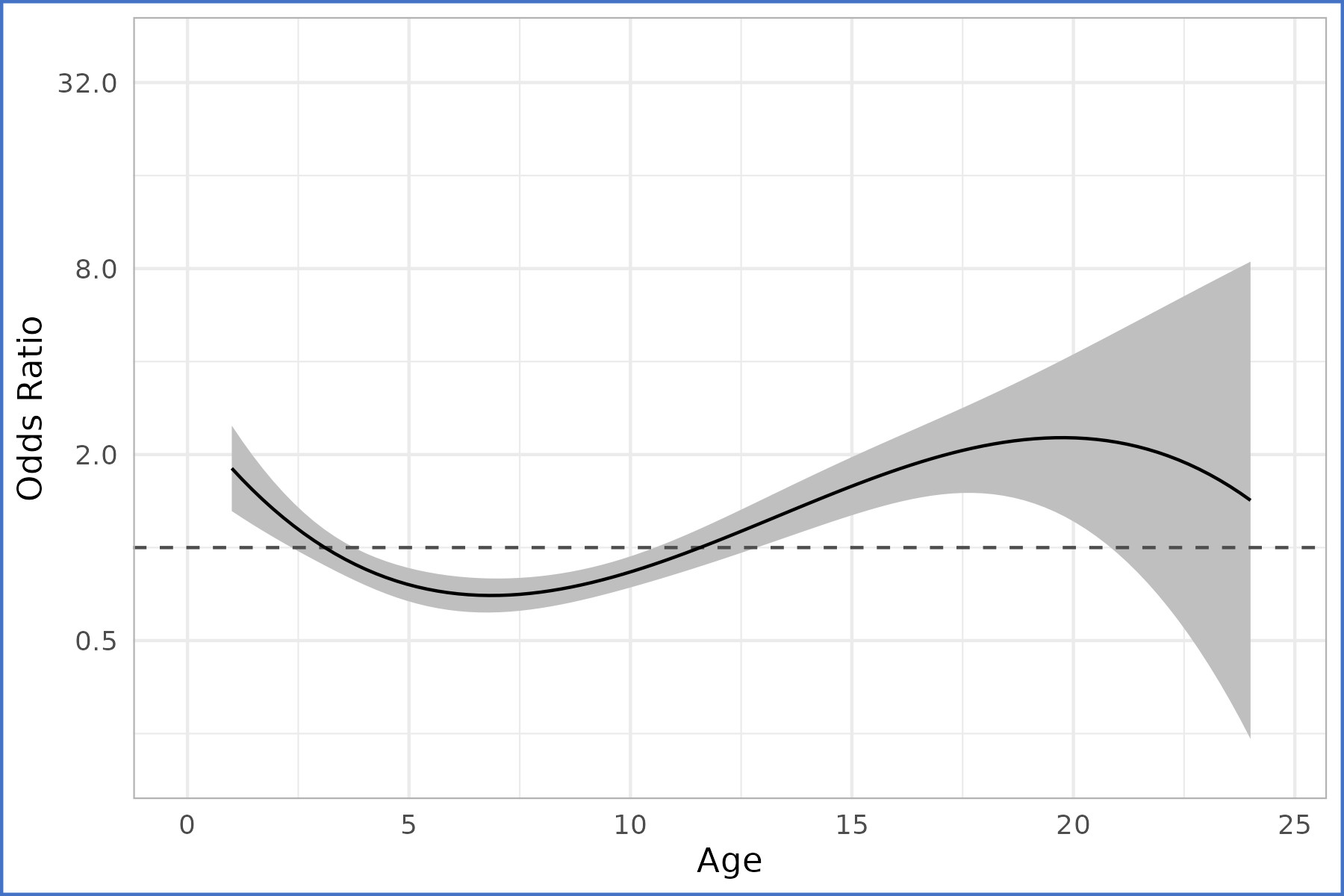

Supplement: S2 Fig — Note: The solid line marks the exponentiated effect estimate, and the grey interval indicates exponentiated 95% point-wise confidence intervals. (TIF) [file pone.0284132.s002.tif]

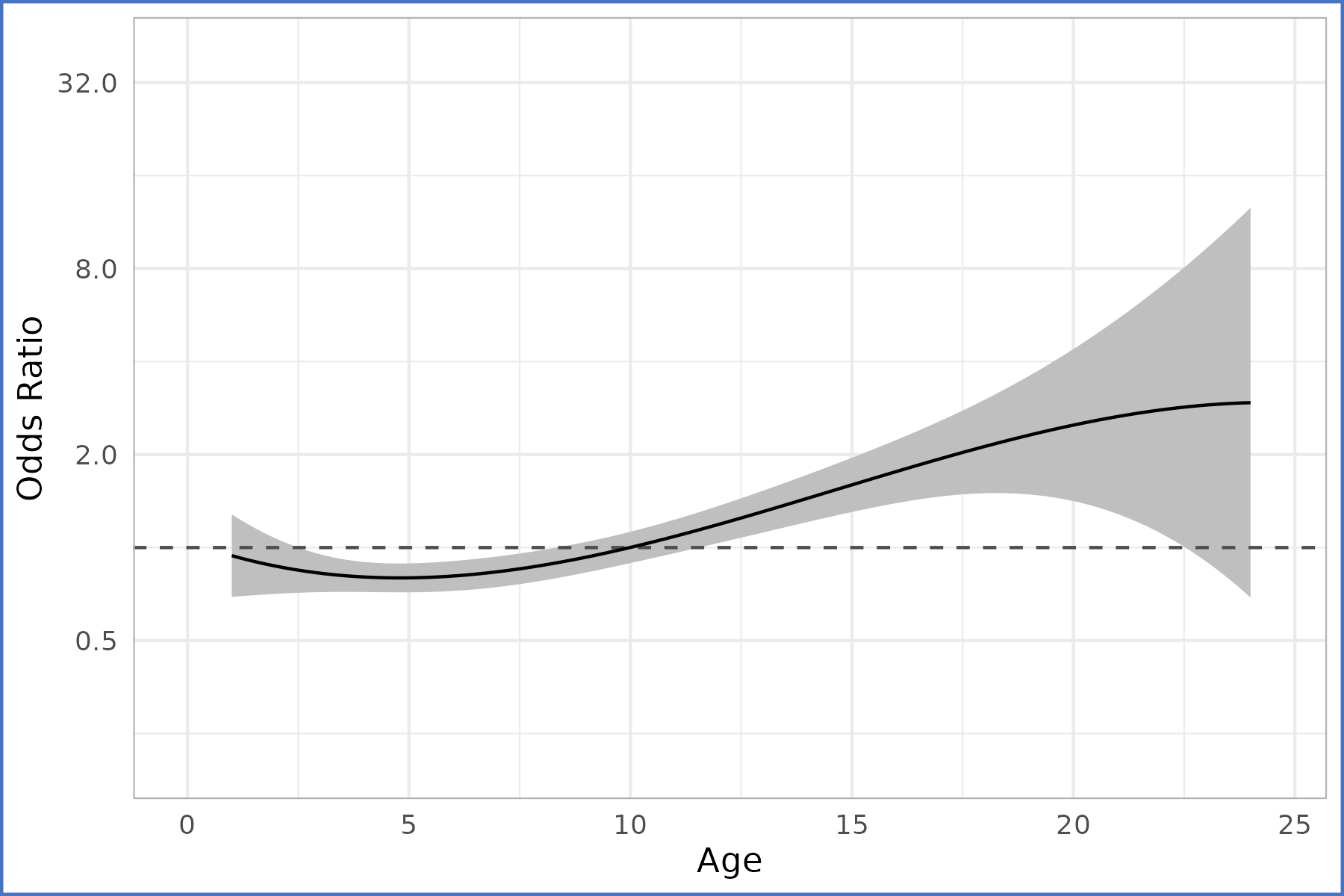

Supplement: S3 Fig — Note: The solid line marks the exponentiated effect estimate, and the grey interval indicates exponentiated 95% point-wise confidence intervals. (TIF) [file pone.0284132.s003.tif]

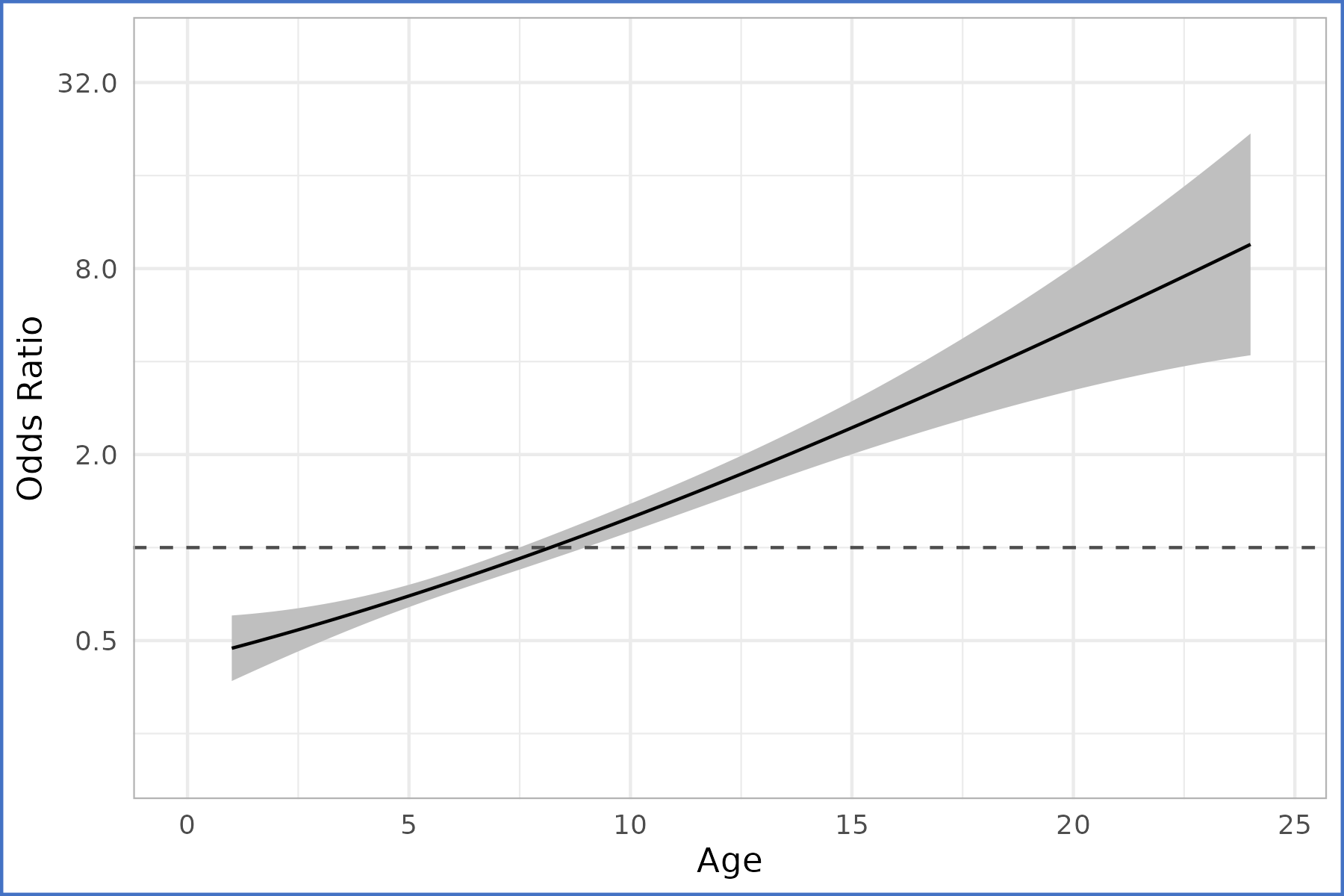

Supplement: S4 Fig — Note: The solid line marks the exponentiated effect estimate, and the grey interval indicates exponentiated 95% point-wise confidence intervals. (TIF) [file pone.0284132.s004.tif]

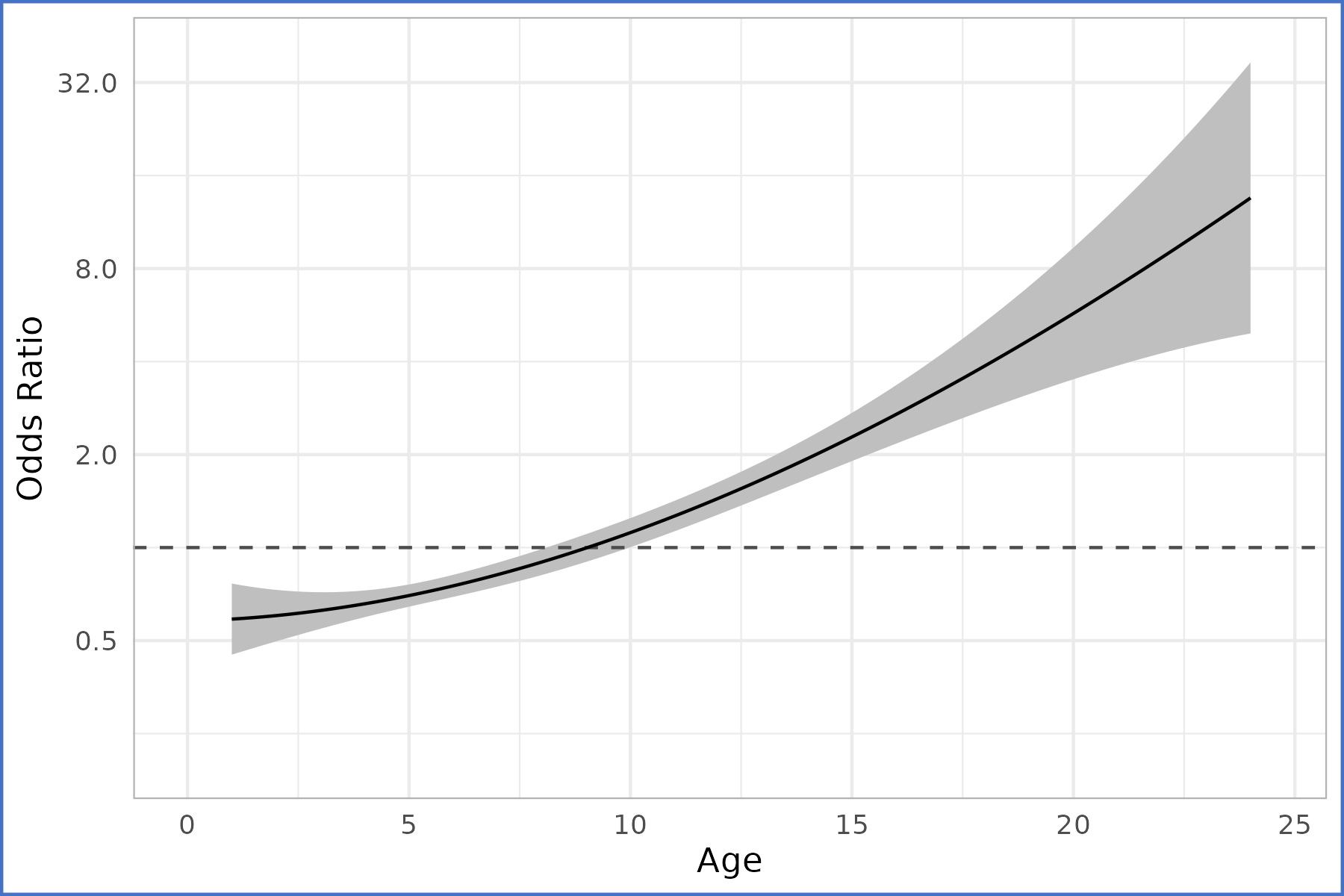

Supplement: S5 Fig — Note: The solid line marks the exponentiated effect estimate, and the grey interval indicates exponentiated 95% point-wise confidence intervals. (TIF) [file pone.0284132.s005.tif]

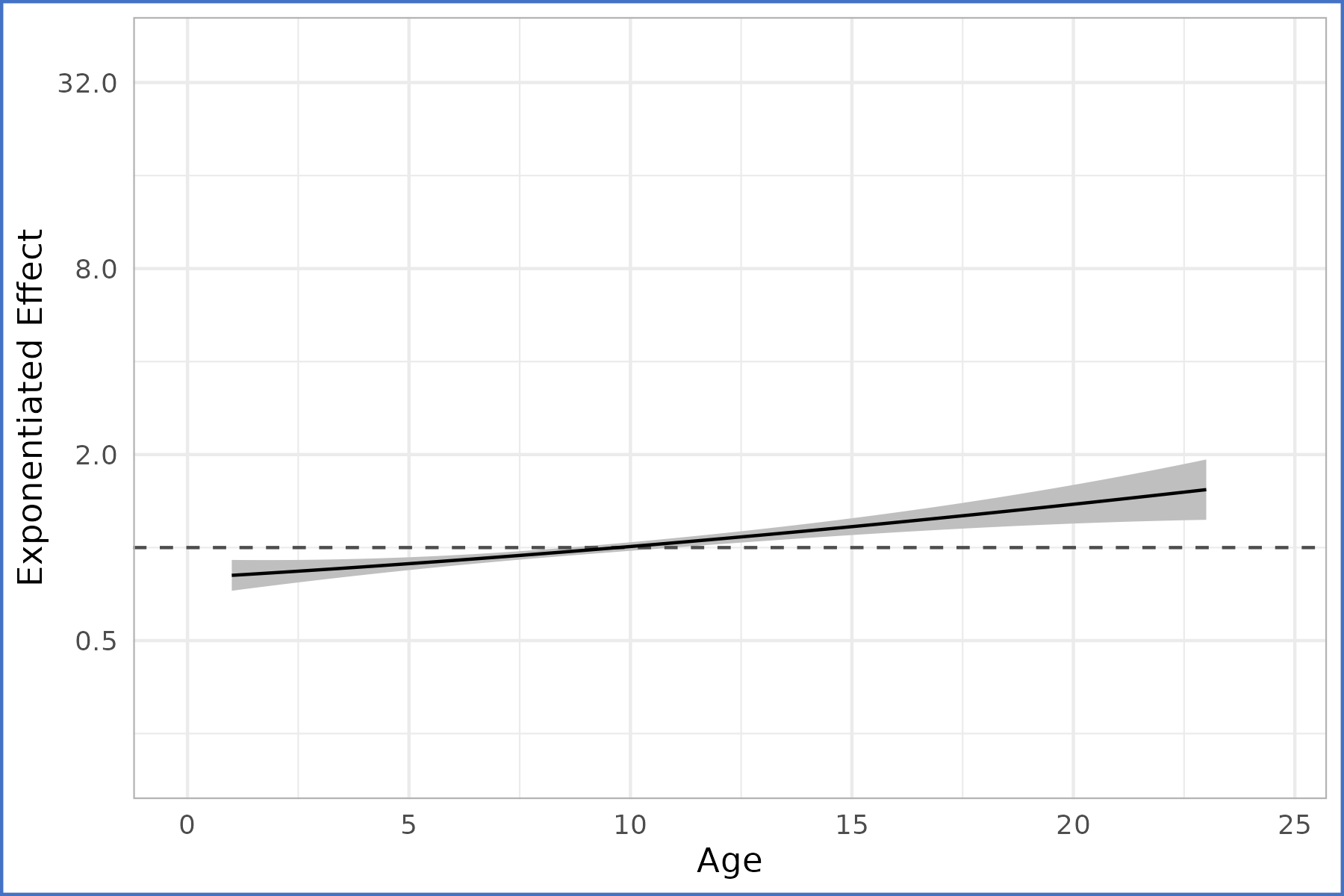

Supplement: S6 Fig — Note: The solid line marks the exponentiated effect estimate, and the grey interval indicates exponentiated 95% point-wise confidence intervals. (TIF) [file pone.0284132.s006.tif]
